# Supplementary material for: MERS-related coronavirus circulating in pangolins exhibits strong fusogenicity in human cells and high sensitivity to fusion inhibitors
Source: Cell Rep Med. 2025 Aug 6;6(8):102277. doi: 10.1016/j.xcrm.2025.102277 (PMC12432378; doi:10.1016/j.xcrm.2025.102277)
Supplement: Document S1. Figures S1–S7 and Tables S1–S3 [file mmc1.pdf]

Cell Reports Medicine, Volume *6*

Supplemental information

MERS-related coronavirus circulating in pangolins exhibits strong fusogenicity in human cells and high sensitivity to fusion inhibitors

Shuai Xia, Fanke Jiao, Jing Chen, Lijue Wang, Tianyu Lu, Qian Wang, Wei Xu, Xinling Wang, Fei Sun, Yun Zhu, Peng Zhou, Shibo Jiang, and Lu Lu

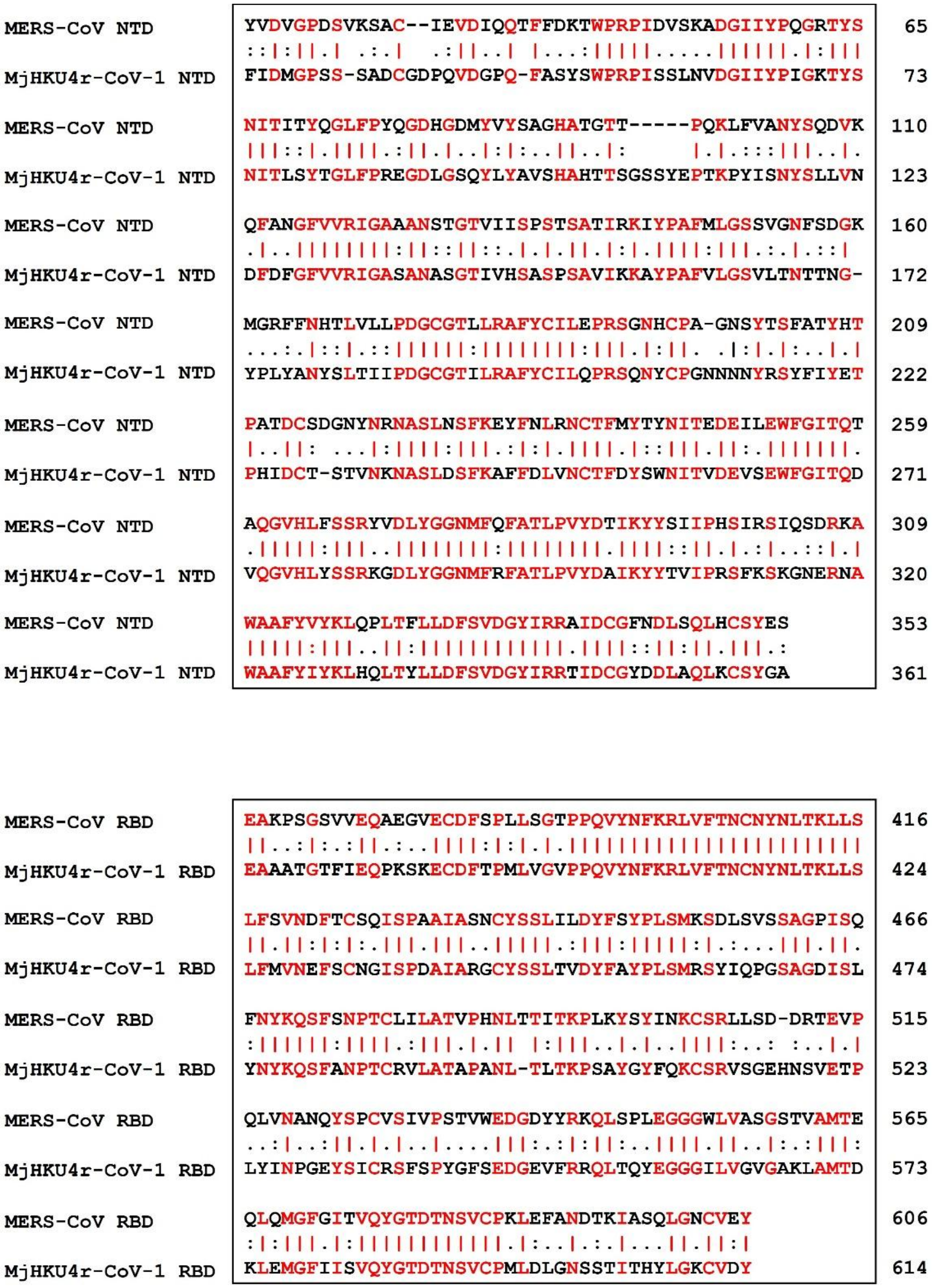

Figure S1. **Sequence similarities between the NTD/RBD domains in S1 of MjHKU4r-CoV-1 and those of MERS-CoV**. Identical amino acid residues are highlighted in red. Related to Figure 1A.

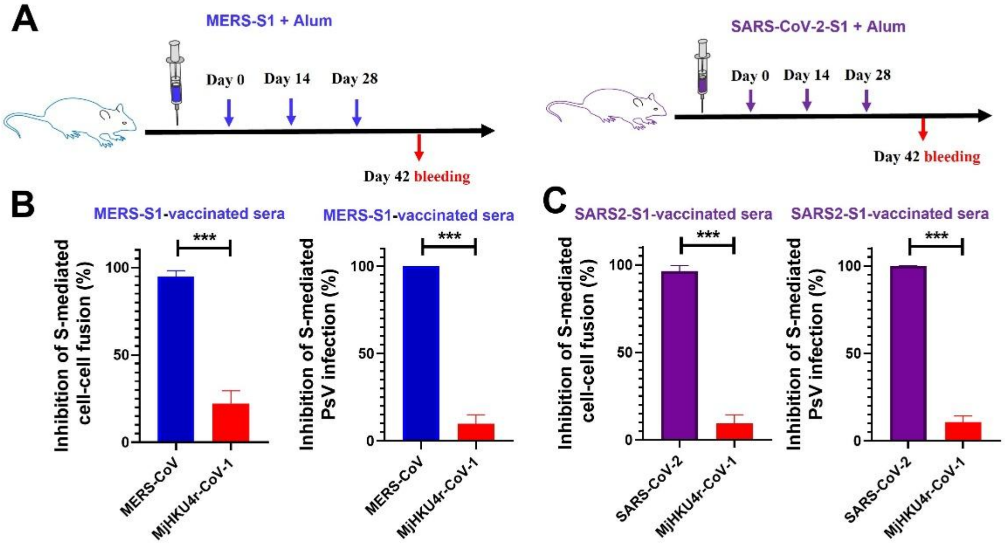

Figure S2. Efficacy of MERS-CoV- or SARS-CoV-2 S1-immunized mouse serum (at 1:300 dilution) against MjHKU4r-S-mediated cell-cell fusion or pseudovirus infection. Data are represented as mean ±SEM of triplicate samples from a representative experiment of at least two independent experiments. p value is from a two-tailed unpaired t-test (***$p<0.001$). Related to Figure 1.

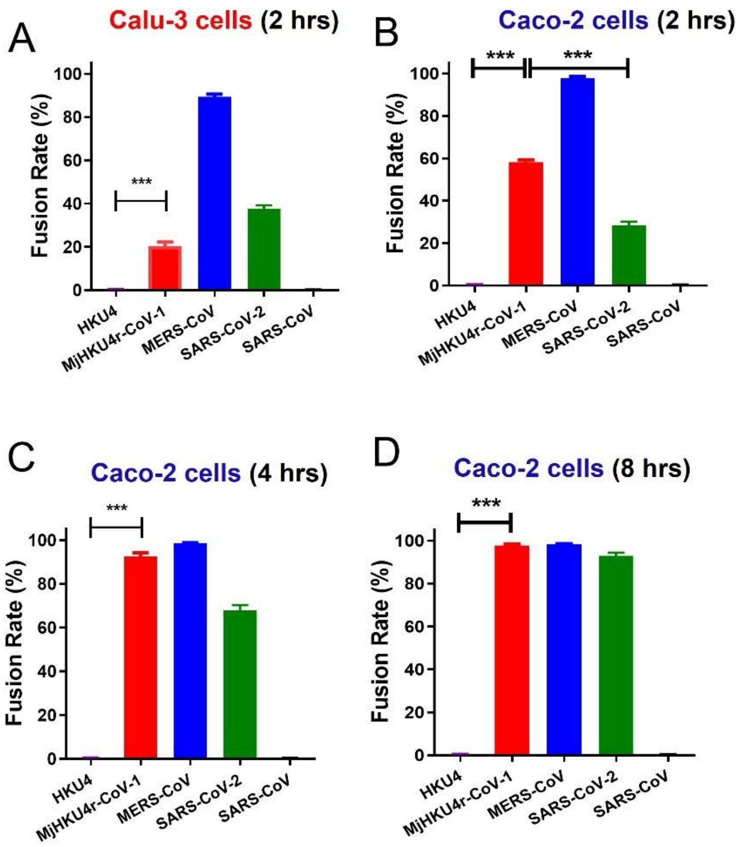

Figure S3. Fusion rates evaluation of MjHKU4-S on Caco-2 cells. Related to Figure 1.

(A-B) Fusion rates mediated by HKU4, MjHKU4-CoV-1, MERS-CoV, SARS-CoV-2 (KP.2) and SARS-CoV S proteins on Calu-3 cells (A) or on Caco-2 cells (B) after coculture for 2 hrs. **(C-D)** Fusion rates mediated by HKU4, MjHKU4-CoV-1, MERS-CoV, SARS-CoV-2 (KP.2) and SARS-CoV S protein on Caco-2 cells after coculture for 4 hrs (C) or 8 hrs (D). Data are represented as mean ±SEM of triplicate samples from a representative experiment of at least two independent experiments. p value is from a two-tailed unpaired t-test (***$p<0.001$). Related to Figure 1.

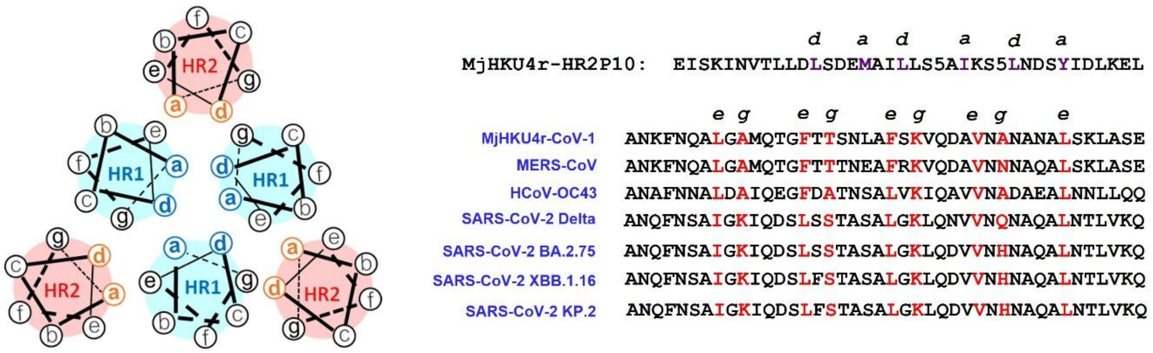

Figure S4. Illustration of 6-HB formation by the HR1 and HR2 domains in the S2 subunit of the MjHKU4r-CoV-1 S protein, along with the mechanism of MjHKU4r-HR2P10 targeting the viral HR1 domain. Related to Figures 3 and 6.

The HR1 trimer is formed by interactions between the amino acid residues (mostly hydrophobic) at the "*a*" and "*d*" positions in the helix wheel of adjacent HR1 domains. This trimer subsequently binds three HR2 domains (or three MjHKU4r-HR2P10 molecules) through interactions with the exposed hydrophobic grooves on the HR1 trimer. Specifically, this binding occurs via interactions between the hydrophobic amino acid residues at the "*a*" and "*d*" positions in the helix wheel of HR2 domains (or MjHKU4r-HR2P10) and those at the "*e*" and "*g*" positions in the helix wheel of HR1 domains.

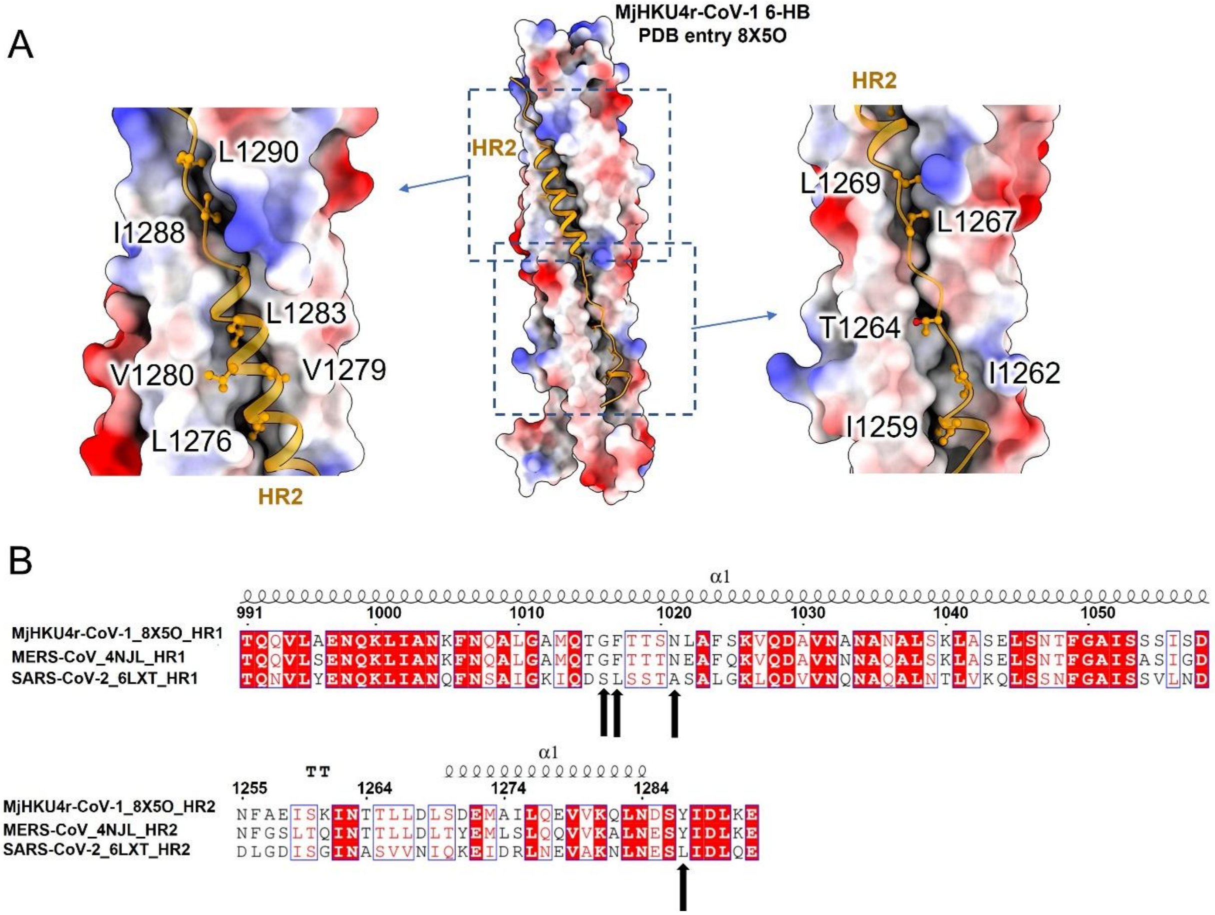

Figure S5. Structural and sequence details of the MjHKU4r 6-HB fusion machine. Related to Figure 4.

(A) The interactions between HR1 and HR2 domains within the hydrophobic grooves of HR1 are displayed in a zoomed-in view and labeled. The hydrophobic grooves of the HR1 trimer are shown as surface electrostatic potential. (B) Multiple sequence alignment of the conserved regions in the HR1 and HR2 domains of the S proteins of MjHKU4r-CoV-1, MERS-CoV, and SARS-CoV-2. Important residues are marked with black arrowheads.

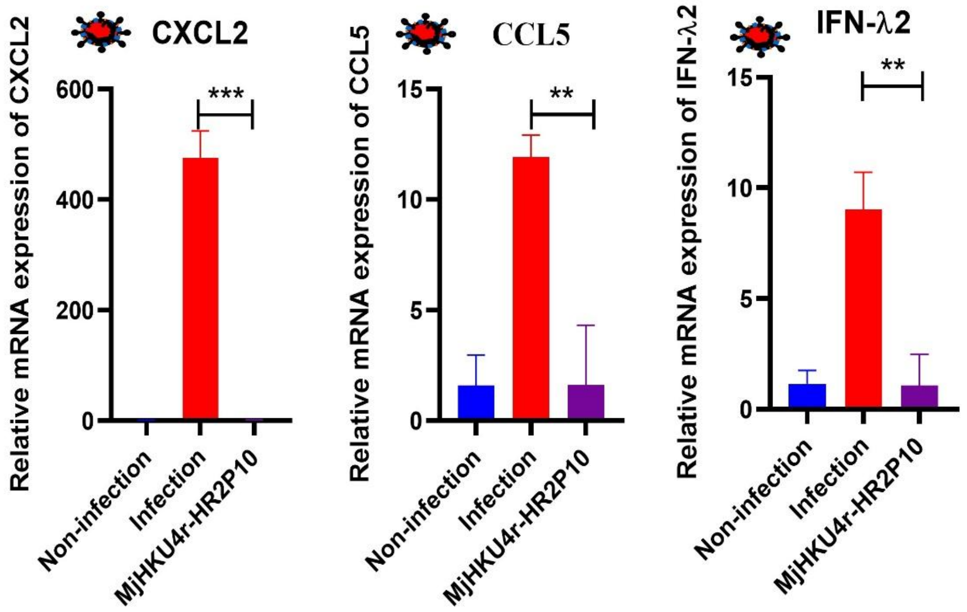

Figure S6. MjHKU4r-HR2P10 at 40 nM completely blocked the upregulation of inflammatory genes (CXCL2, CCL5, IFN-λ2) induced by MjHKU4r-CoV-1 infection in Caco-2 cells. Data are represented as mean ±SEM of triplicate samples from a representative experiment of at least two independent experiments. p value is from a two-tailed unpaired t-test (**p<0.01, ***p<0.001). Related to Figure 6.

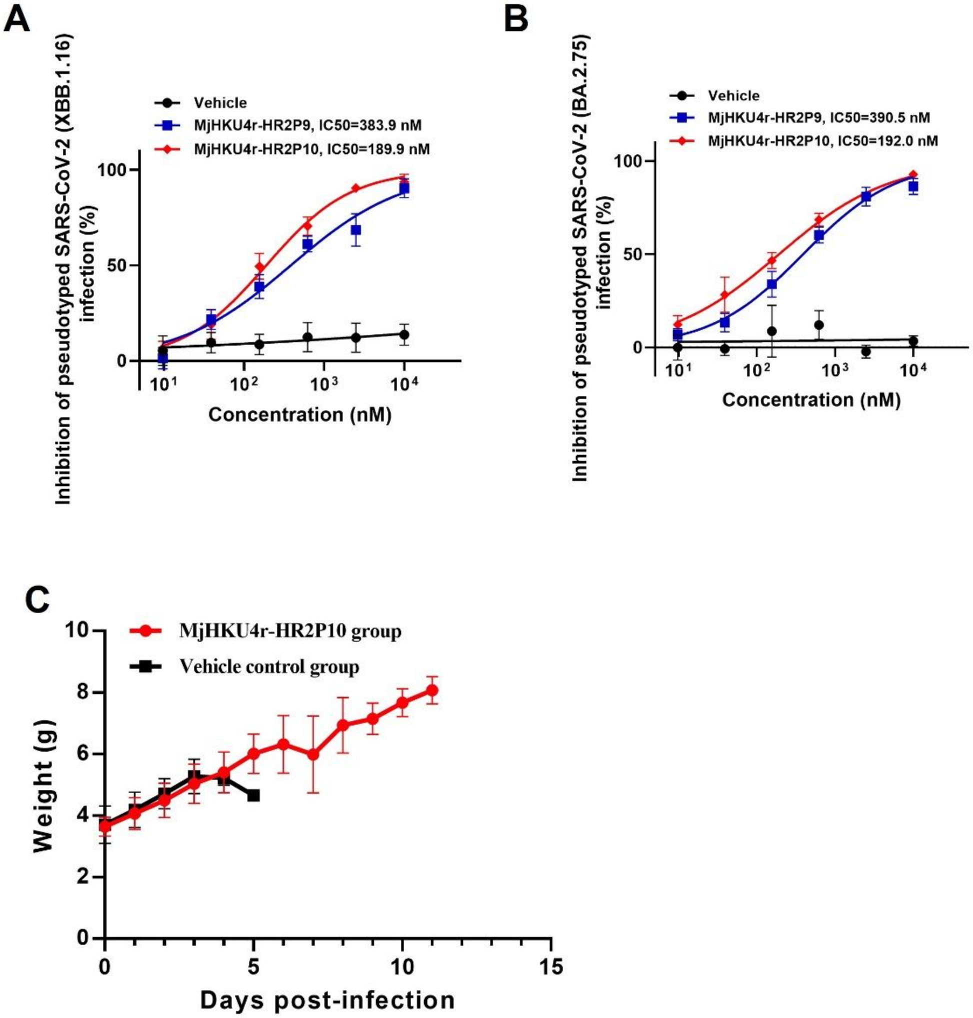

Figure S7. Inhibitory efficacy of MjHKU4r-HR2P10 against pseudotyped SARS-CoV-2-XBB.1.16 (A) and SARS-CoV-2-BA.2.75 (B) infections, and the body weight change of newborn mice treated with MjHKU4r-HR2P10 after HCoV-OC43 challenge (C). Data are represented as mean ±SEM. Related to Figure 7.

Table S1. The sequence of MjHKU4r-HR2P to MjHKU4r-HR2P10, related to Figure 6.

Peptides	Sequence
**MjHKU4r-HR2P**	EISKINTTLLDLSDEMAILQEVVKQLNDSYIDLKEL
**MjHKU4r-HR2P2**	PNFAEISKINTTLLDLSDEMAILQEVVKQLNDSYIDLKEL
**MjHKU4r-HR2P3**	EISKINTTLLDLSDEMAILQEVVKQLNDSLIDLKEL
**MjHKU4r-HR2P4**	EISKINTTLLDLSDEMAILQEAIKQLNDSYIDLKEL
**MjHKU4r-HR2P5**	EISKINVTLLDLSDEMAILQEVVKQLNDSYIDLKEL
**MjHKU4r-HR2P6**	EISKINVTFLDLSDEMAILQEVVKQLNDSYIDLKEL
**MjHKU4r-HR2P7**	EISKINTTLLDLSDEMAILLEVVKQLNDSYIDLKEL
**MjHKU4r-HR2P8**	EISKINTTLLDLSDFMAILLEVVKQLNDSYIDLKEL
**MjHKU4r-HR2P9**	EISKINVTLLDLSDEMAILLEAIKQLNDSYIDLKEL
**MjHKU4r-HR2P10**	EISKINVTLLDLSDEMAILL-S5-AIK-S5-LNDSYIDLKEL

Table S2. Data collection and refinement statistics, related to Figure 4.

	MjHKU4r HR1-HR2<br>PDB entry 8X5O
**Data collection**	
Space group	P 63 2 2
Cell dimensions	
a, b, c (Å)	198.7, 198.7, 64.1
α, β, γ (°)	90, 90, 120
Wavelength (Å)	1.5418
Resolution (Å)	34.42 -2.67 (2.80 - 2.67) †
R_{merge}	0.43 (2.11)
Mean I/σ(I)	12.5 (2.5)
Completeness (%)	99.5 (97.0)
Redundancy	37.6 (37.3)
**Refinement**	
Resolution (Å)	33.12 -2.67
No. of reflections	21595
Reflections in test set	1094
R_{work}/R_{free}	0.241/0.292
No. of protein atoms	
	2489
Water & Ligands	0
Root mean square (rms) deviations	
Bond lengths (Å)	0.003
Bond angles (°)	0.49
Ramachandran Outliers(%)	0
Average *B*-factor (Å^2)	38.58

†Highest resolution shell is shown in parenthesis.

Table S3. The sequences of primers for RT-qPCR, related to STAR Methods.

Primer	Sequence
**IFN-β1**	5′-AACTGCAACCTTTCGAAGCC-3′ (forward)<br>5′-TGTCGCCTACTACCTGTTGTGC-3′ (reverse)
**TNF-α**	5′-GAGGCCAAGCCCTGGTATG-3′ (forward)<br>5′-CGGGCCGATTGATCTCAGC-3′ (reverse)
**IL-6**	5′-CCAGGAGAAGATTCCAAAGATGTA-3′ (forward)<br>5′-CGTCGAGGATGTACCGAATTT-3′ (reverse)
**CCL5**	5′-TGCCCACATCAAGGAGTATTT-3′ (forward)<br>5′-GATGTACTCCCGAACCCATTT-3′ (reverse)
**CXCL2**	5′-GCTTGTCTCAACCCCGCATC-3′ (forward)<br>5′-TGGATTTGCCATTTTTCAGCATCTT-3′ (reverse)
**IFN-λ1**	5′-CCTCACGCGAGACCTCAAAT-3′ (forward)<br>5′-GAGTAGGGCTCAGCGCATAA-3′ (reverse)
**IFN-λ2**	5′-AATTGTGTTGCCAGTGGGGA-3′ (forward)<br>5′-GCGACTGGGTGGCAATAAAT-3′ (reverse)
**IFN-λ3**	5′-CCCAAAAAAGGAGTCCCCTG-3′ (forward)<br>5′-GGTTGCATGACTGGCGGA-3′ (reverse)
**GAPDH**	5′- GTCTCCTCTGACTTCAACAGCG-3′ (forward)<br>5′- ACCACCCTGTTGCTGTAGCCAA -3′ (reverse)
