# Supplementary material for: MERS-related coronavirus circulating in pangolins exhibits strong fusogenicity in human cells and high sensitivity to fusion inhibitors
Source: Cell Rep Med. 2025 Aug 6;6(8):102277. doi: 10.1016/j.xcrm.2025.102277 (PMC12432378; doi:10.1016/j.xcrm.2025.102277)
Supplement: Document S2. Article plus supplemental information [file mmc2.pdf]

Cell Reports
Medicine

Article

MERS-related coronavirus circulating in pangolins exhibits strong fusogenicity in human cells and high sensitivity to fusion inhibitors

Graphical abstract

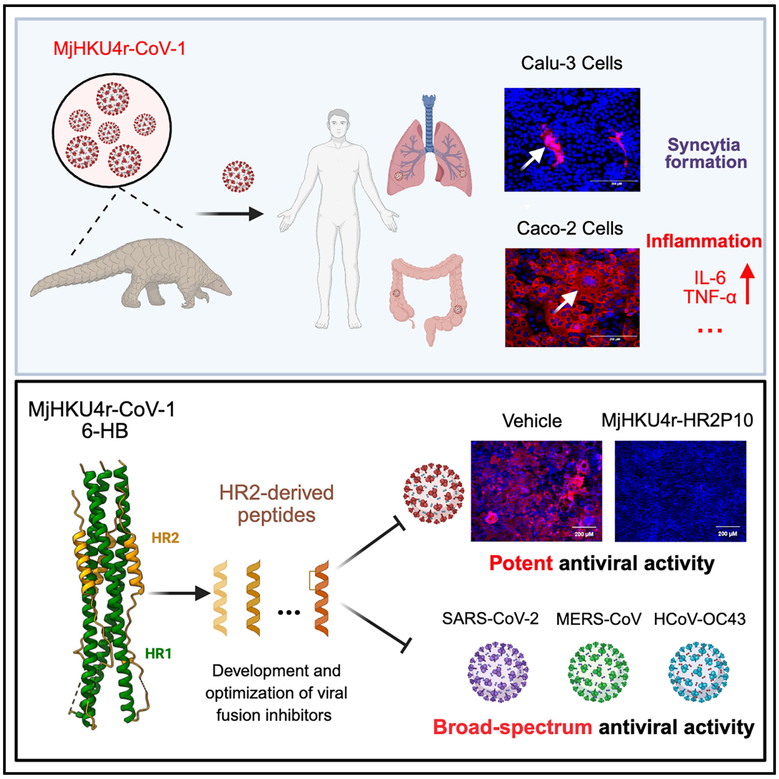

Highlights

- MjHKU4r-CoV-1 with high fusogenicity induces inflammatory responses in human cells
- 6-HB structure determination unveils MjHKU4r-S-mediated membrane fusion mechanism
- MjHKU4r-CoV-1 HR2 peptides exhibit potent activity by targeting viral HR1 domain
- Stapled peptide MjHKU4r-HR2P10 shows potent and broad-spectrum anti-CoV activity

Authors

Shuai Xia, Fanke Jiao, Jing Chen, ..., Peng Zhou, Shibo Jiang, Lu Lu

Correspondence

sxia15@fudan.edu.cn (S.X.),
zhuyun@ibp.ac.cn (Y.Z.),
zhou_peng@gzlab.ac.cn (P.Z.),
shibojiang@fudan.edu.cn (S.J.),
lul@fudan.edu.cn (L.L.)

In brief

Xia et al. reveal that MjHKU4r-CoV-1 has high fusogenicity and can induce inflammatory responses in human cells. By determining the six-helix bundle (6-HB) structure of MjHKU4r-S, they clarify its membrane fusion mechanism and develop HR1-targeting fusion inhibitors with potent antiviral activity.

Xia et al., 2025, Cell Reports Medicine 6, 102277
August 19, 2025 © 2025 The Author(s). Published by Elsevier Inc.
https://doi.org/10.1016/j.xcrm.2025.102277

CellPress

Cell Reports Medicine

CellPress
OPEN ACCESS

Article

MERS-related coronavirus circulating in pangolins exhibits strong fusogenicity in human cells and high sensitivity to fusion inhibitors

Shuai Xia,[1,4,*] Fanke Jiao,[1,4] Jing Chen,[2,4] Lijue Wang,[1,4] Tianyu Lu,[1,4] Qian Wang,[1] Wei Xu,[1] Xinling Wang,[1] Fei Sun,[3] Yun Zhu,[3,*] Peng Zhou,[2,*] Shibo Jiang,[1,*] and Lu Lu[1,5,*]

[1]Shanghai Public Health Clinical Center, Key Laboratory of Medical Molecular Virology (MOE/NHC/CAMS), Shanghai Institute of Infectious Disease and Biosecurity, School of Basic Medical Sciences, Shanghai Frontiers Science Center of Pathogenic Microbes and Infection, Fudan University, Shanghai, China

[2]Guangzhou Laboratory, No. 9 Xing Dao Huan Bei Road, Guangzhou International Bio Island, Guangzhou, Guangdong Province 51005, China

[3]National Key Laboratory of Biomacromolecules, CAS Center for Excellence in Biomacromolecules, Institute of Biophysics, Chinese Academy of Sciences, Beijing, China

[4]These authors contributed equally

[5]Lead contact

*Correspondence: sxia15@fudan.edu.cn (S.X.), zhuyun@ibp.ac.cn (Y.Z.), zhou_peng@gzlab.ac.cn (P.Z.), shibojiang@fudan.edu.cn (S.J.), lul@fudan.edu.cn (L.L.)

https://doi.org/10.1016/j.xcrm.2025.102277

SUMMARY

Unlike preceding MERS-related coronaviruses, the recently identified MjHKU4r-CoV-1 strain can directly infect human cells. Nonetheless, its potential pathogenic attributes and underlying molecular mechanisms remain unclear. We find that MjHKU4r-CoV-1 induces significant inflammation, including interleukin (IL)-6 and tumor necrosis factor alpha (TNF-α), and exhibits pronounced fusogenicity mediated by its spike (S) protein, leading to extensive syncytium formation. This suggests the possibility that MjHKU4r-CoV-1 possesses strong pathogenic potential in humans. Further, we successfully reveal the molecular mechanism of MjHKU4r-S-driven membrane fusion by crystallizing the six-helix bundle (6-HB) structure, a fusion apparatus composed of HR1 and HR2 domains. Concurrently, we develop a series of peptide-based fusion inhibitors that target the viral HR1 domain to impede the formation of viral 6-HB. Among these fusion inhibitors, a stapled peptide, MjHKU4r-HR2P10, shows the most potent inhibitory activity against MjHKU4r-CoV-1, MERS-CoV, SARS-CoV-2, and HCoV-OC43 infections at nanomolar level and thus holds considerable promise for further development as effective antiviral agents in clinic.

INTRODUCTION

Coronaviruses (CoVs) are ubiquitous in nature, hosting a diverse range of intermediary species and presenting a substantial threat to human health.[1] The Middle East respiratory syndrome coronavirus (MERS-CoV), identified in 2012, rapidly disseminated across multiple nations.[2] Characterized by its utilization of human dipeptidyl peptidase-4 (hDPP4) as a cellular receptor,[3] MERS-CoV exhibits potent fusogenicity and alarmingly high pathogenicity.[4,5] To date, MERS-CoV has resulted in 2,609 infections with a mortality rate of 36%,[6] significantly exceeding the 0.9% mortality rate observed in the ongoing COVID-19 pandemic (https://covid19.who.int/). This stark contrast underscores the imminent threat posed by Middle East respiratory virus (MERS)-related CoVs, including bat-CoV HKU4, bat-CoV HKU5-1/2, and MOW15-22/PnNL2018B.[7–11] In fact, these prior MERS-related CoVs demonstrated limited infectivity in human cells and lacked clearly identified intermediate hosts for their evolutionary transition. However, the recent discovery of the *Manis javanica* HKU4-related CoV (MjHKU4r-CoV-1) in Malayan pangolins signals a potential shift.[12] Similar to MERS-CoV, MjHKU4r-CoV-1 also exhibits high affinity to its receptor, hDPP4.[12] Furthermore, MjHKU4r-CoV-1 demonstrates significant infectivity in human cells, human organs, and hDPP4-transgenic mice.[12] Serological assessments indicated that approximately 12.8% of Malayan pangolins in Southeast Asia are positive for MjHKU4r-CoV-1,[12] suggesting a significant evolutionary foothold and the potential for future cross-species transmission to humans. However, its specific pathogenic features and underlying mechanisms remain unclear. This deficit in bench science has, so far, forestalled our identification of optimal viral targets and the development of potent antiviral interventions to effectively combat any possible epidemic or pandemic caused by MjHKU4r-CoV-1 or its descendant lineages.

The CoV spike (S) protein plays a pivotal role in viral infection and cross-species transmission.[13] Hence, S-mediated cellular fusogenicity is a key virological feature closely associated with viral infectivity and pathogenesis,[14,15] and MERS-CoV demonstrates

© 2025 The Author(s). Published by Elsevier Inc.
This is an open access article under the CC BY license (http://creativecommons.org/licenses/by/4.0/).

potent fusogenicity along with high virulence *in vivo*.[4] As further evidence of S-mediated cellular fusogenicity, autopsies of COVID-19 patients revealed the presence of syncytial dysmorphic pneumocytes in lung tissue, resulting from S-mediated cellular fusion between infected cells and adjacent target cells.[16] Such syncytia destroy normal cellular function and life cycle, inducing secondary pathological inflammatory responses.[17] Severe SARS-CoV-2 infections are believed to be directly associated with inflammatory cytokine storm with interleukin (IL)-6, tumor necrosis factor alpha (TNF-α) expressed at abnormally high levels.[18–22] Additionally, S-mediated cellular fusion facilitates virus transmission, effectively evading humoral immunity and antibody-based therapeutics.[23,24] Considering its infectivity in human cells, it is imperative to explore the virological features driven by the MjHKU4r-CoV-1 S protein (MjHKU4r-S), which was identified as having exceptionally high fusogenicity, and develop efficient countermeasures.

During CoV infection, receptor-S protein engagement triggers conformational changes in the viral S2 subunit leading to the formation of a six-helix bundle (6-HB). This structure plays a crucial role in bringing viral and cellular membranes closer together and facilitating membrane fusion.[4] Structurally, however, 6-HB varies among different CoVs, leading to significant differences in their fusogenicity.[25] Such variation makes it imperative to study the specific structure and function of 6-HB in MjHKU4r-CoV-1. Such study will form the foundation for the development of anti-MjHKU4r-CoV-1 therapeutics.

Based on our MjHKU4r-CoV-1 S-mediated cell-cell fusion model, we found that MjHKU4r-S has remarkably evolved fusion capacity able to trigger an even more significant inflammatory response when compared to the current SARS-CoV-2. In the course of developing our model, we elucidated the structural composition of its 6-HB fusion apparatus, which consists of three heptad repeat (HR)1 and HR2 domains, providing the critical clues toward understanding the molecular mechanism underlying S-driven membrane fusion. We then successfully developed MjHKU4r-HR2-derived peptides, which demonstrated significant efficacy in inhibiting MjHKU4r-CoV-1 infection with half-maximal inhibitory concentrations (IC_{50}s) in the nanomolar range. In particular, among these peptides, a stapled peptide, MjHKU4r-HR2P10, showed the most potent antiviral activity inhibiting MjHKU4r-CoV-1 infection and broadly preventing MERS-CoV, SARS-CoV-2, and HCoV-OC43 infections. These results suggest that the HR1 domain of MjHKU4r-CoV-1 is an ideal therapeutic target and that these peptide-based fusion inhibitors could be further developed into clinical antiviral agents against MjHKU4r-CoV-1 and other emerging MERS-related CoVs to combat future epidemic or pandemic caused by MjHKU4r-CoV-1 or its descendant lineages.

RESULTS

MjHKU4r-CoV-1 features remarkable inflammation and fusogenicity in human cells

The CoV S protein plays a crucial role in mediating viral fusion, infection, and pathogenicity. However, functional characteristics of the MjHKU4r-CoV-1 S protein have not been elucidated. Here, we systematically evaluated the sequence identity of functional domains in the MjHKU4r-CoV-1 S protein in comparison with those in MERS-CoV S protein.[26] We found that MjHKU4r-S exhibited about 65.7% identity with MERS-CoV-S, particularly a 73.3% identity in their respective S2 subunits (Figure 1A). In the S1 subunit, the N-terminal domain (NTD) of MjHKU4r-S displayed 54.8% identity with MERS-CoV-S NTD (Figures 1A and S1). Despite sharing the same hDPP4 receptor, their respective receptor-binding domains (RBDs) only showed 58.9% identity (Figures 1A and S1). When we immunized mice with MERS-CoV or SARS-CoV-2 S1 protein and evaluated the inhibitory activity of the mouse sera against MjHKU4r-CoV-1 infection, we found that neither anti-MERS-CoV nor anti-SARS-CoV-2 S1 sera were effective against MjHKU4r-CoV-1 pseudovirus (PsV) infection (Figure S2). These results showed the daunting challenge in developing MERS-CoV-S-targeted vaccines and neutralizing antibody drugs against infection of MjHKU4r-CoV-1 and its descendant lineages.

Remarkably, live MjHKU4r-CoV-1 infection induced a pronounced syncytium phenomenon in both human Calu-3 lung cells and Caco-2 colon cells (Figures 1B and 1C), indicating the potent fusion capacity of MjHKU4r-CoV-1 S protein. Meanwhile, we observed that authentic MjHKU4r-CoV-1 infection in Caco-2 cells significantly induced inflammation, with substantial regulation of gene expression, including IL-6, TNF-α, interferon (IFN)-β1, IFN-λ1, and IFN-λ3 (Figure 1D), suggesting that MjHKU4r-CoV-1 possesses high pathogenic potential in humans. After developing an S-mediated cell-cell fusion system for MjHKU4r-CoV-1, we were surprised to find that MjHKU4r-S effectively mediated about 20% fusion in Calu-3 cells at the 2-h mark, exceeding that of HKU4-S with 0% fusion at the same time (Figures 1C and S3A). Meanwhile, MjHKU4r-S drove its fusion capacity on Caco-2 to 58.2% at the 2-h time point (Figures 1C and S3B), which was significantly higher than that of the currently circulating SARS-CoV-2 S protein (28.4% fusion). At the 4-h or 8-h time points, MjHKU4r-S-mediated fusion tended toward saturation, similar to that in the MERS-CoV-S group (Figures S3C and S3D). Fusion kinetics assessments revealed that MjHKU4r-CoV-1 S protein mediated higher fusion efficiency than that of SARS-CoV-2 and HKU4 on Caco-2 cells (Figure 1E).

MjHKU4r-S-mediated cell fusion triggers robust inflammatory responses

Syncytial formation, a key pathological feature observed in SARS-CoV-2 infection, significantly exacerbates disease severity in COVID-19 patients.[16] To investigate the transcriptional response induced by MjHKU4r-S-mediated syncytium formation, we performed RNA sequencing (RNA-seq) and quantitative reverse-transcription PCR (RT-qPCR) analyses to evaluate transcriptomic changes and identify related pathways. Effector cells expressing HKU4-S or SARS-CoV-2-S protein served as negative or positive control, respectively (Figure 2A). In both MjHKU4r-CoV-1 and SARS-CoV-2 compared with HKU4, gene set enrichment analysis (GSEA) demonstrated significant enrichment of immune response pathways, including TNF-α signaling via nuclear factor κB (NF-κB), the inflammatory response pathway, and IFN response (IFN-γ and IFN-α) with normalized enrichment score (NES) > 2 and adjusted p values (P.adj) < 0.05 (Figure 2B). Notably, MjHKU4r-CoV-1

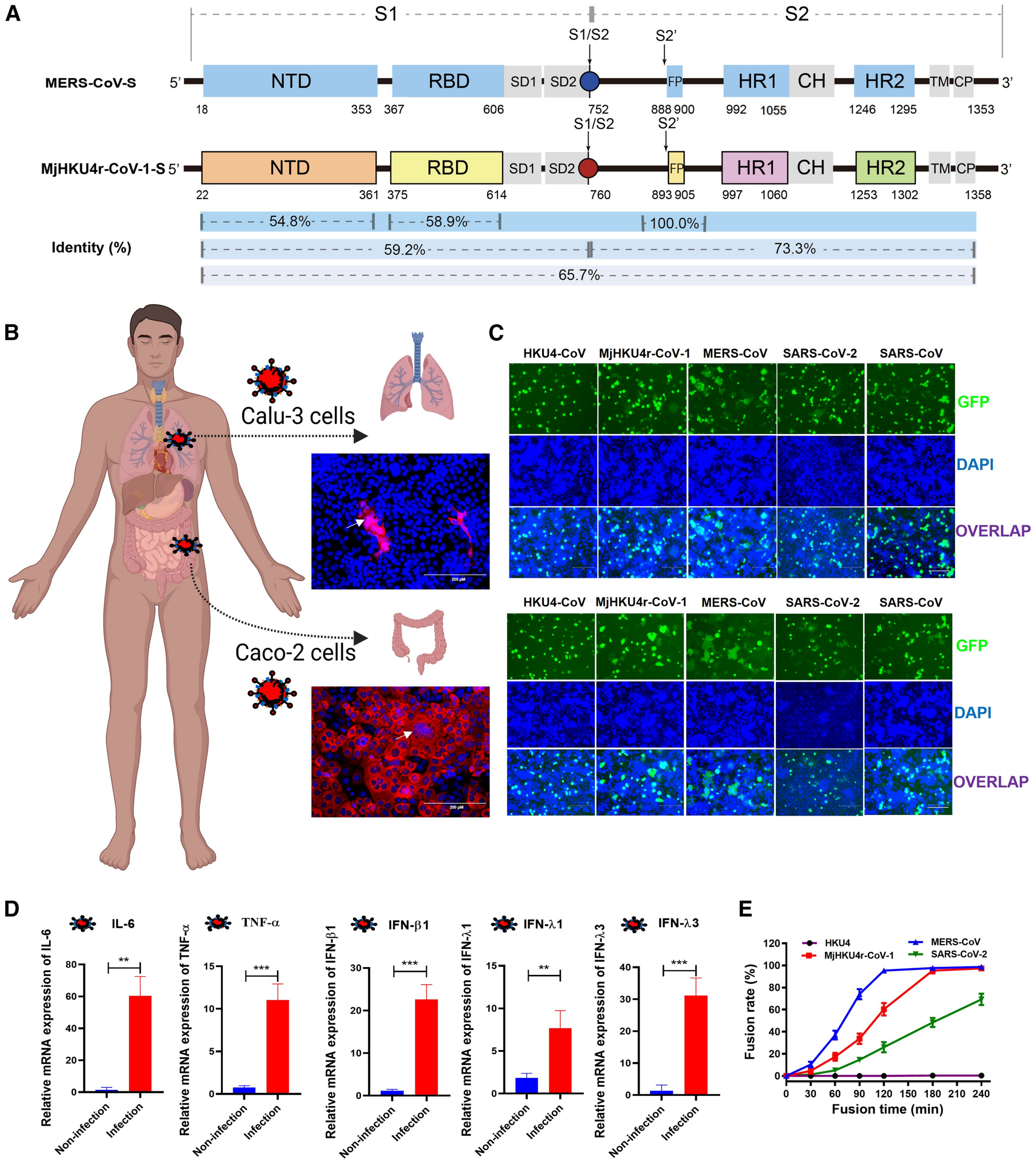

Figure 1. Potent cellular fusogenicity feature mediated by MjHKU4r-S

(A) Schematic representation of MjHKU4r-CoV-1 S protein with residue identity in comparison with MERS-CoV S protein. Its S1 subunit contains NTD (22–361 aa) and RBD (375–614 aa), and its S2 subunit contains FP (893–905 aa), HR1 (997–1,060 aa), and HR2 (1,253–1,302 aa).

(B) Formation of syncytium in Calu-3 and Caco-2 cells 48 h after authentic MjHKU4r-CoV-1 infection through immunofluorescence staining. Arrows indicate syncytia. Scale bars, 200 μm. Red, nucleocapsid protein (NP); blue, nuclei.

(C) Representative images of HKU4-, MjHKU4r-CoV-1-, MERS-CoV-, SARS-CoV-2 (KP.2)-, and SARS-CoV S-mediated cell-cell fusion on Calu-3 cells (upper) and Caco-2 cells (lower) at 2 h. Scale bars, 150 μm. Blue, nuclei.

(D) Relative mRNA expression levels of IL-6, TNF-α, IFN-β1, IFN-λ1, and IFN-λ3 in Caco-2 cells 48 h after authentic MjHKU4r-CoV-1 infection, all quantified via RT-qPCR.

(E) Fusion kinetics evaluation of HKU4, MjHKU4-CoV-1, MERS-CoV, and SARS-CoV-2 (KP.2) S proteins on Caco-2 cells.

Data are represented as mean ± SEM of triplicate samples from a representative experiment of at least two independent experiments. *p* value is from a two-tailed unpaired t test (**$p < 0.01$, ***$p < 0.001$).

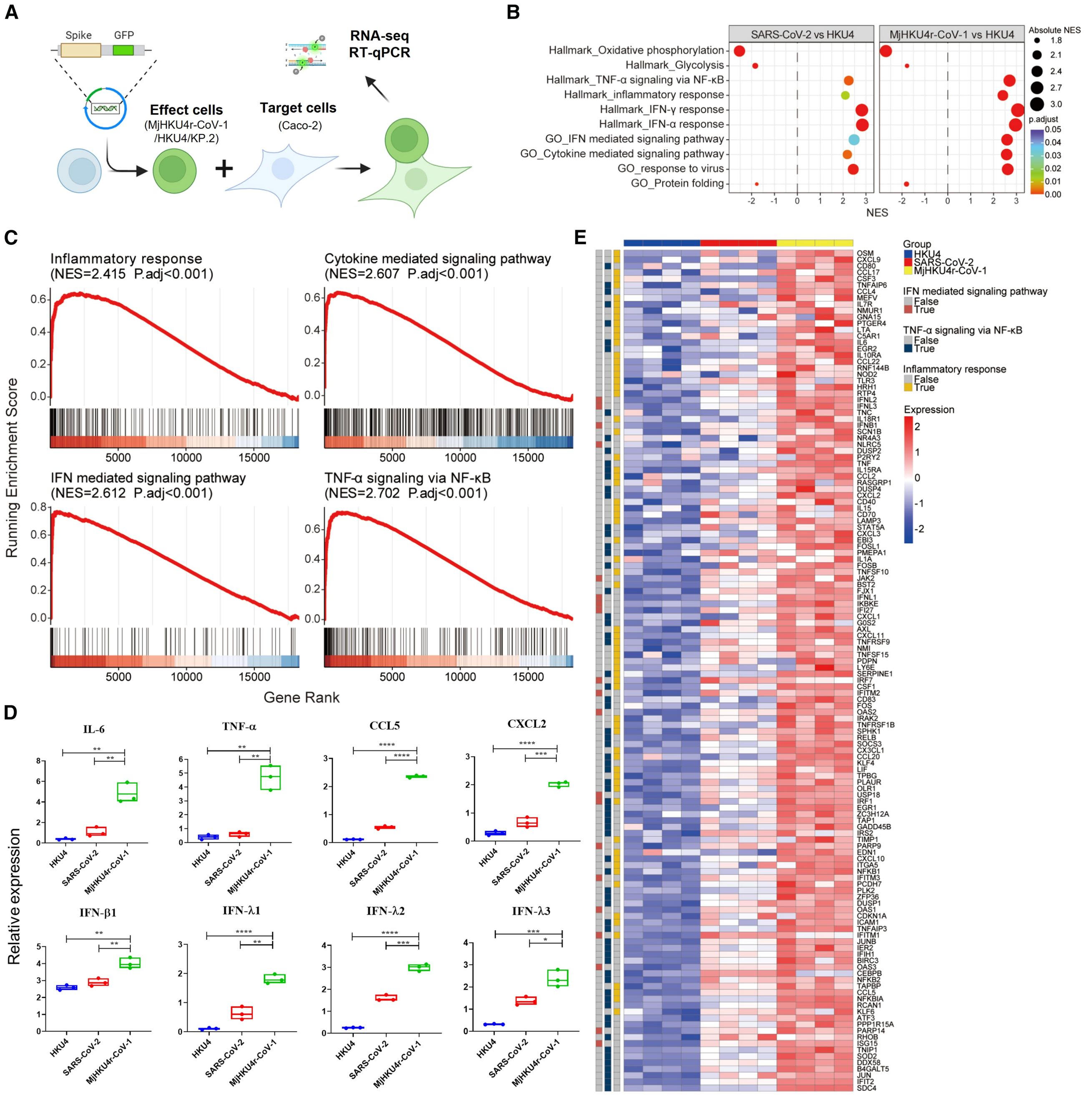

Figure 2. MjHKU4r-S-mediated cell fusion triggers substantial inflammatory responses

(A) Schematic illustration of cell-cell fusion driven by the S proteins of HKU4, MjHKU4r-CoV-1, or SARS-CoV-2 (KP.2).

(B) GSEA results showing significant pathways enriched in target cells fused with effector cells expressing SARS-CoV-2 or MjHKU4r-S proteins, compared to those fused with HKU4-S effector cells. The *x* axis indicates NES values, while the *y* axis lists significant pathways. Dot size represents absolute NES values, and dot color indicates P.adj significant levels.

(C) GSEA plots comparing MjHKU4r-S and HKU4-S for such pathways as inflammatory response, cytokine-mediated signaling, interferon (IFN)-mediated signaling, and TNF-α signaling via NF-κB. NES and P.adj values are provided for each pathway. The *x* axis represents genes ranked by logFC, and the *y* axis shows running enrichment scores. Bottom vertical lines indicate genes associated with these pathways.

(D) Relative mRNA expression levels of IL-6, TNF-α, CCL5, CXCL2, IFN-β1, IFN-λ1, IFN-λ2, and IFN-λ3 genes in HKU4, SARS-CoV-2 (KP.2), and MjHKU4r-CoV-1 groups, all quantified via RT-qPCR. Data are represented as mean ± SEM of triplicate samples from a representative experiment of at least two independent experiments. *p* value is from a two-tailed unpaired t test (***p* < 0.01, ****p* < 0.001, *****p* < 0.0001).

(E) Heatmap showing the relative expression of core-enrichment genes in these pathways for each group. Pathways, group details, and expression level references are annotated to the right, while pathway-associated genes are noted on the left. Heatmap grid colors represent relative expression levels.

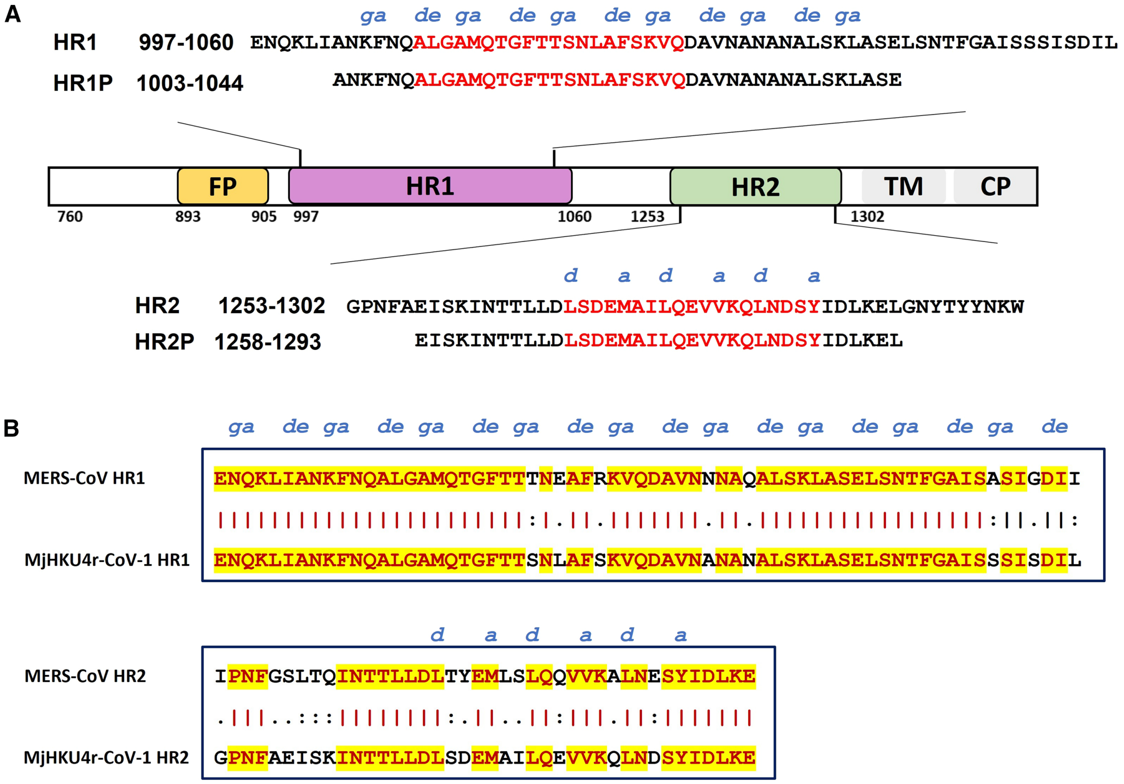

Figure 3. The functional domains in the MjHKU4r S protein S2 subunit and amino acid sequences of peptides derived from the HR1 and HR2 domains

(A) Schematic representation of MjHKU4r S protein S2 subunit. Residue numbers of each region correspond to their positions in S protein of MjHKU4r-CoV-1. Corresponding sequences of MjHKU4r-HR1P and MjHKU4r-HR2P.

(B) Sequence similarities between HR1 domain (residues 997–1,060) in S2 of MjHKU4r-CoV-1 and that of MERS-CoV (residues 992–1,055) and N-terminal portion of the HR2 domain (residues 1,253–1,292) in S2 of MjHKU4r-CoV-1 and that of MERS-CoV (residues 1,246–1,285). Identical amino acid residues are highlighted in red, with yellow background.

showed stronger activation of these pathways compared to HKU4 and even SARS-CoV-2 (Figure 2B).

Detailed enrichment plots for selected immune and inflammatory pathways highlighted MjHKU4r's robust signaling. For example, IFN-mediated signaling (NES = 2.612) and cytokine-mediated signaling (NES = 2.607) pathways were prominently activated. Additionally, TNF-α signaling via NF-κB (NES = 2.702) and the inflammatory response pathway (NES = 2.415) were more enriched in MjHKU4r-CoV-1 than those observed in HKU4 (Figure 2C), indicating strong inflammatory regulation in MjHKU4r-CoV-1 samples.

Validation of transcriptomic findings through RT-qPCR analysis further confirmed significant upregulation of inflammation-related genes in MjHKU4r-CoV-1 samples. These included IL-6, TNF-α, CCL5, and CXCL2 with expression levels significantly surpassing those observed in SARS-CoV-2 and HKU4 controls (Figure 2D), which were positively related to severity in COVID-19 patients.[20] IFN-related genes, including IFN-β1, IFN-λ1, IFN-λ2, and IFN-λ3, were also substantially upregulated in MjHKU4r-CoV-1 samples (Figure 2D). The unique expression patterns of immune- and inflammation-related genes in the MjHKU4r-S group underscore its distinct pathogenic profile (Figure 2E). Taken together, these findings demonstrate that MjHKU4r-CoV-1 samples exhibit significant enrichment of inflammation-related pathways, suggesting that MjHKU4r-CoV-1 possesses higher pathogenic potential in human intestinal epithelial tissue relative to that of the current SARS-CoV-2.

The overall structure of the MjHKU4r-CoV-1 6-HB fusion machine is highlighted by its 6-HB fusion core

It is well known that the 6-HB fusion machine drives viral fusion. Based on sequence alignment, we located the specific region of HR1 (residues 997–1,060) and HR2 (residues 1,253–1,302), the fusion cores of HR1 and HR2 spanning 1,009–1,028 and 1,269–1,287 residues, respectively (Figure 3A). Compared with MERS-CoV, these two functional domains of MjHKU4r-CoV-1 possessed moderate amino acid identity of 87.5% and 74% between HR1 and HR2, respectively. HR1 and HR2 have 8 and 13 different residues, respectively (Figure 3B). Based on the heptad repeat sequence feature of MERS-CoV reported previously,[4] we herein systematically located these critical residues at the ***a*** and ***d*** positions or ***e*** and ***g*** positions in the HR1 helix participating in the interaction with another HR1 helix or with

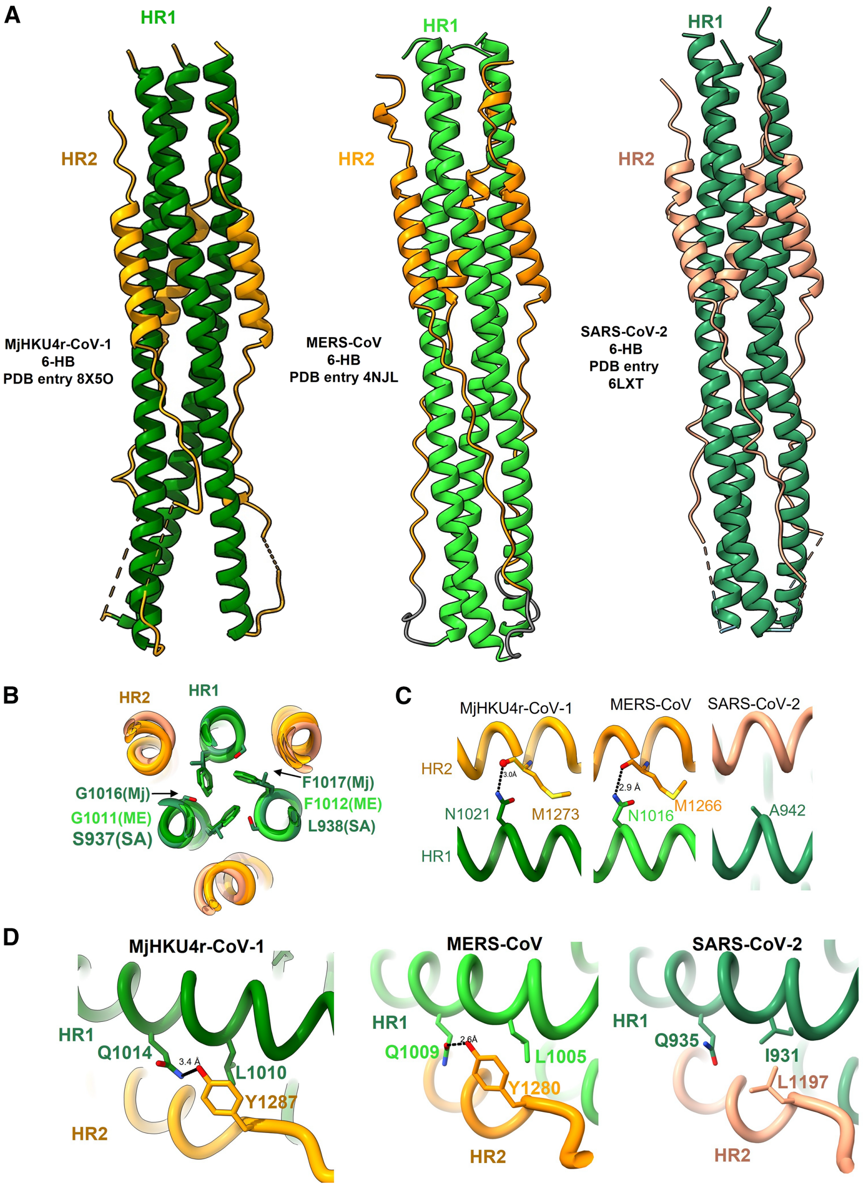

Figure 4. Crystal structure of MjHKU4r-6-HB

(A) The crystal structures of 6-HB from MjHKU4r-CoV-1, MERS-CoV, and SARS-CoV-2 are shown in cartoon representation. Their HR1/HR2 motifs are colored in green/orange, lime green/dark orange, and sea green/light salmon, respectively.

(B) Superimposed structures of 6-HB from MjHKU4r-CoV-1 (Mj), MERS-CoV (ME), and SARS-CoV-2 (SA). Important residues are highlighted as sticks in the zoom-in view.

(C and D) Parallel comparison of 6-HB from MjHKU4r-CoV-1, MERS-CoV, and SARS-CoV-2. Important residues are highlighted as sticks in the zoom-in view.

HR2 helix, respectively, as well as residues at the ***a*** and ***d*** positions in HR2 helix involved in interaction with HR1 helices (Figures 3 and S4).

To understand the structural basis of the interactions between HR1 and HR2 regions of MjHKU4r-CoV-1, we constructed and expressed a recombinant protein containing HR1 (residues 989–1,064) and HR2 (residues 1,253–1,296) with a short linker (SGGRGG) for crystallographic study. Then we determined the crystal structure of the MjHKU4r-CoV-1 spike protein post-fusion core (Figure 4A). It reveals that the HR1 and HR2 domains of MjHKU4r-CoV-1 adopt the classical 6-HB conformation. The three HR1 domains are intertwined by forming a central triple helix bundle through hydrophobic interactions. Then, the grooves on the HR1 trimer are embraced by the inversely parallel HR2 domain in triple helix arrangement (Figure 4A). In the HR2 region, from S1270 to S1286, a regular helical structure is formed, while its two sides adopt an extended linear conformation. This overall structure resembles that of MERS-CoV and SARS-CoV-2.

Upon further examining the side-chain conformation of the MjHKU4r-CoV-1 6-HB residue, we uncovered intriguing insights into the affinity between HR1 and HR2. Specifically, numerous hydrophobic amino acids in HR2 participate in binding with the hydrophobic groove of HR1, including I1259, I1262, T1264, L1267, L1269, L1276, V1279, V1280, L1283, I1288, and L1290 (Figure S5A). Since the HR2 motif is in close proximity to HR1, many oxygen and nitrogen atoms on the HR1 main chain form interactions with side chains of residues on HR1, including N998, K1005, N1021, K1026, N1032, and N1034. Furthermore, several residues on HR2 engage in charge interactions with side chains of HR1, such as E1272-K1026, S1286-Q1008, and K1291-E997. Additionally, it appears that the interaction strength of the MjHKU4r-CoV-1 6-HB fusion machine is similar to that of MERS-CoV, possibly even stronger than that of SARS-CoV-2. For instance, three Phe residues establish hydrophobic interactions within the HR1 trimer in both MjHKU4r-CoV-1 (F1017) and MERS-CoV (F1012). In contrast, SARS-CoV-2 substitutes this Phe with Leu938, and its neighboring Gly is replaced by Ser937, which may affect the hydrophobicity of HR1 trimer to some extent (Figures 4B and S5B). Furthermore, in MjHKU4r-CoV-1, Asn1021 in HR1 forms interactions with the main chain carbonyl oxygen of Met1273 in HR2, mirroring the behavior observed in MERS-CoV. However, this Asn residue is substituted with Ala 942 in SARS-CoV-2, resulting in the loss of such interactions at this site (Figure 4C). Moreover, in MjHKU4r-CoV-1, Tyr1287 engages in hydrophobic interactions and hydrogen bonding with Leu1010 and Gln1014 of HR1, respectively, in a manner reminiscent of MERS-CoV. However, this Tyr

in HR2 of SARS-CoV-2 S protein S2 subunit is replaced with Leu1197, thereby severing the hydrogen bond with its HR1 (Figure 4D).

MjHKU4r-CoV-1 HR2-derived peptide demonstrates significant biophysical activity and potent antiviral activity

Mimicry of the viral HR1 and HR2 domains was established by conducting biophysical characterization of the HR1 and HR2 peptides of MjHKU4r-CoV-1. According to the distribution of these key residues, we rationally designed HR1-derived peptide MjHKU4r-HR1P (residues 1,003–1,044) and HR2-derived peptide MjHKU4r-HR2P (residues 1,258–1,293) (Figure 3A). In non-denaturing polyacrylamide gel electrophoresis (N-PAGE), MjHKU4r-HR1P, possessing positive charges, did not form a band, whereas MjHKU4r-HR2P, with negative charges, produced a distinct lower band (Figure 5A). However, the MjHKU4r-HR1P/MjHKU4r-HR2P mixture exhibited a new upper band (Figure 5A), demonstrating the interaction between MjHKU4r HR1 and HR2 domains in mediating viral fusion and entry.

Meanwhile, circular dichroism (CD) spectroscopy was employed to assess the secondary structures of MjHKU4r-HR1P and MjHKU4r-HR2P and their complex. As shown in Figure 5B, MjHKU4r-HR1P alone predominantly exhibited a random coil structure, whereas MjHKU4r-HR2P displayed a lower α-helicity of 18.2%. The MjHKU4r-HR1P/MjHKU4r-HR2P mixture, however, showed a high α-helicity of 76.9% characterized by a saddle-shaped negative peak and a remarkable molar ellipticity increase at 222 nm (Figure 5B), further indicating that MjHKU4r-HR1P and MjHKU4r-HR2P could mimic the viral HR1 and HR2 domains, thereby forming a helical fusion machine. Moreover, this helical complex demonstrated strong thermal stability with a melting temperature (Tm) of 84.8°C (Figure 5C). Thus, stability of the helical complex was comparable to that of MERS-HR1P/MERS-HR2P complex (87°C)[4] but substantially higher than that of the SARS-CoV-2-HR1P/SARS-CoV-2-HR2P complex (66.2°C).[27] These results further show the stability of the 6-HB fusion machine, which, in turn, supports the potent fusion capacity of MjHKU4r S protein.

Considering the significant biophysical activity of MjHKU4r-CoV-1 HR-derived peptides, we further evaluated their fusion-inhibitory efficacy. We found that MjHKU4r- HR2P at the concentration of 2,500 nM completely inhibited MjHKU4r-S-mediated cell-cell fusion (Figure 5D). Further quantitative evaluation showed that the IC_{50} of MjHKU4r-HR2P against MjHKU4r-S-driven cell fusion was 36.99 nM, while MjHKU4r-HR1P exhibited no significant inhibition, even at concentrations up to 10,000 nM (Figure 5E). Consistently, MjHKU4r-HR2P could effectively inhibit MjHKU4r-CoV-1 PsV infection with an IC_{50} of 61.99 nM, but MjHKU4r-HR1P could not (Figure 5F). When we applied the authentic MjHKU4r-CoV-1 infection system, MjHKU4r-HR2P also demonstrated potent antiviral activity with an IC_{50} of 26.02 nM (Figure 5G). In line with data from the RT-qPCR assay, the results from immunofluorescence staining showed near-complete blockage of MjHKU4r-CoV-1 N protein expression in Caco-2 cells at concentrations of 5,000 nM and 1,000 nM MjHKU4r-HR2P (Figure 5H), further suggesting the potential of HR2-derived peptides as antiviral agents against MjHKU4r-CoV-1 infection.

Optimized HR2 peptides show increased efficacy against MjHKU4r-CoV-1 and other β-HCoV infections

Based on the structure of MjHKU4r-6-HB and our prior experience with peptide optimization, we designed some derivatives of MjHKU4r-HR2P (Table S1; Figure 6A). Among these derivatives, MjHKU4r-HR2P9 demonstrated significantly increased fusion-inhibitory potency with an IC_{50} value of 10.27 nM (Figure 6B). In the next iteration, MjHKU4r-HR2P10, we introduced a pair of (S)-2-(4-pentenyl)alanine (S5) substitutions at positions 1278E and 1282Q. This modification created an intramolecular hydrocarbon staple, reinforcing the bioactive secondary structure, and further improving antiviral efficacy (Figure 6A).[28] As anticipated, MjHKU4r-HR2P10 exhibited the most potent fusion-inhibitory activity with an IC_{50} of 3.56 nM (Figure 6B). MjHKU4r-HR2P9 and MjHKU4r-HR2P10 also displayed robust inhibitory effects against pseudotyped MjHKU4r-CoV-1 infection with IC_{50} values of 25.96 nM and 8.29 nM, respectively, representing a respective 2.9-fold and 9.1-fold improvement compared to inhibitory effects of the original MjHKU4r-HR2P (Figure 6C). Notably, MjHKU4r-HR2P10 at 40 nM almost completely suppressed authentic MjHKU4r-CoV-1 N protein expression (Figure 6D). Consistently, RT-qPCR results showed that MjHKU4r-HR2P10 potently inhibited MjHKU4r-CoV-1 replication with an IC_{50} value of 2.13 nM (Figure 6E). Additionally, MjHKU4r-HR2P10 at 40 nM completely inhibited inflammatory response (Figures 6F and S6). These findings highlight MjHKU4r-HR2P10 as a promising therapeutic candidate with superior antiviral potency, providing a solid foundation for combating potential MjHKU4r-CoV-1 outbreaks.

To further evaluate the broad-spectrum inhibitory activity of MjHKU4r-HR2P10, we examined its cross-inhibitory efficacy against other β-HCoVs. Notably, MjHKU4r-HR2P10 significantly blocked MERS-CoV PsV infection, achieving an IC_{50} of 37.2 nM (Figure 7A). This potency surpasses that of the previously reported MERS-HR2P.[4] More intriguingly, both MjHKU4r-HR2P10 and MjHKU4r-HR2P9 effectively inhibited a range of SARS-CoV-2 variants, including Delta, BA.2.75, XBB.1.16, and KP.2, with IC_{50} values between 85.9 and 192.0 nM for MjHKU4r-HR2P10 and between 383.9 and 459.5 nM for MjHKU4r-HR2P9 (Figures 7B, 7C, S7A, and S7B). Additionally, both peptides exhibited significant inhibitory activity against HCoV-OC43 with IC_{50} values of 479.9 nM for MjHKU4r-HR2P10 and 1,023 nM for MjHKU4r-HR2P9 (Figure 7D). We further assessed the protective efficacy of MjHKU4r-HR2P10 *in vivo* using a lethal HCoV-OC43-infected mouse model. Mice in the vehicle control group exhibited progressive weight loss and 100% mortality within 7 days post-infection (dpi) (Figures 7E, 7F, and S7C). In contrast, mice treated with MjHKU4r-HR2P10 experienced only minimal weight loss following HCoV-OC43 challenge, and rapid recovery was observed by 8 dpi (Figure S7C), finally showing a 66.7% survival rate to further underscore its potent therapeutic efficacy against viral infection *in vivo* (Figures 7E and 7F).

DISCUSSION

MjHKU4r-CoV-1 is a recently identified MERS-related CoV that has been extensively circulating in Malayan pangolins.[12] Like MERS-CoV, it can bind to its hDPP4 receptor and significantly infect human cells,[12] thus posing a significant threat to human

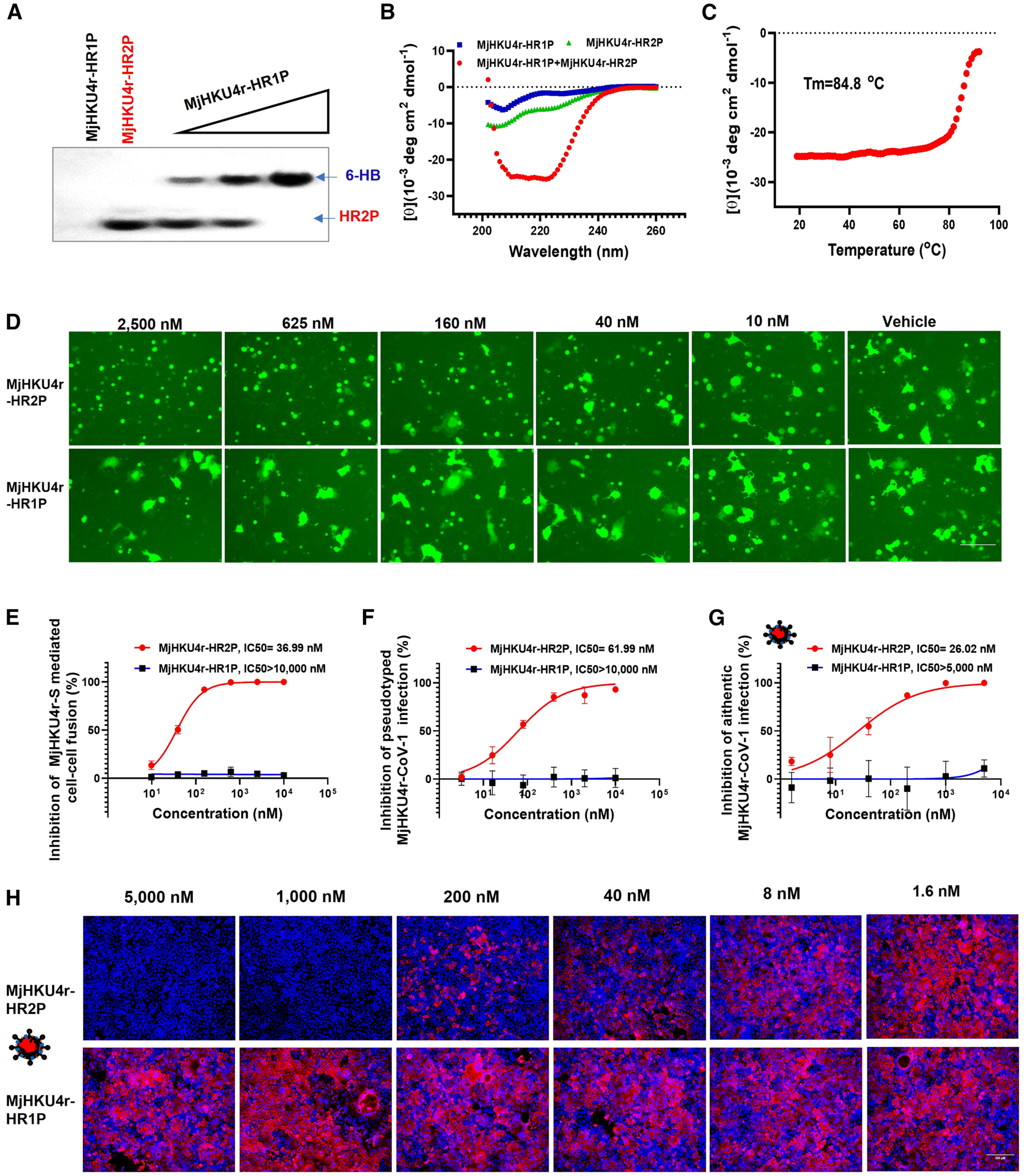

Figure 5. Inhibitory activity of MjHKU4r-HR2P against MjHKU4r-CoV-1 infection

(A and B) The interaction between MjHKU4r-HR1P and MjHKU4r-HR2P in N-PAGE (A) and CD spectra (B).

(C) Tm value of the MjHKU4r-HR1P/MjHKU4r-HR2P complex.

(D) Representative images of cell-cell fusion mediated by MjHKU4r-S on Caco-2 cells after coculture for 4 h in the presence of MjHKU4r-HR1P or MjHKU4r-HR2P with indicated concentration. Scale bars, 150 μm. Blue, nuclei.

(E) Efficacy of MjHKU4r-HR1P or MjHKU4r-HR2P against MjHKU4r-S-mediated cell-cell fusion.

(F) Inhibitory activity of MjHKU4r-HR1P or MjHKU4r-HR2P against MjHKU4r-S-mediated pseudovirus infection.

(G) Inhibitory efficacy of MjHKU4r-HR1P or MjHKU4r-HR2P against authentic MjHKU4r-CoV-1 infection was measured by RT-qPCR.

(H) Immunofluorescence assay to confirm the antiviral activity of MjHKU4r-HR2P against authentic MjHKU4r-CoV-1 with an antibody against the HKU4 N protein (right, scale bars, 200 μm). Red, NP; blue, nuclei.

Data are represented as mean ± SEM of triplicate samples from a representative experiment of at least two independent experiments.

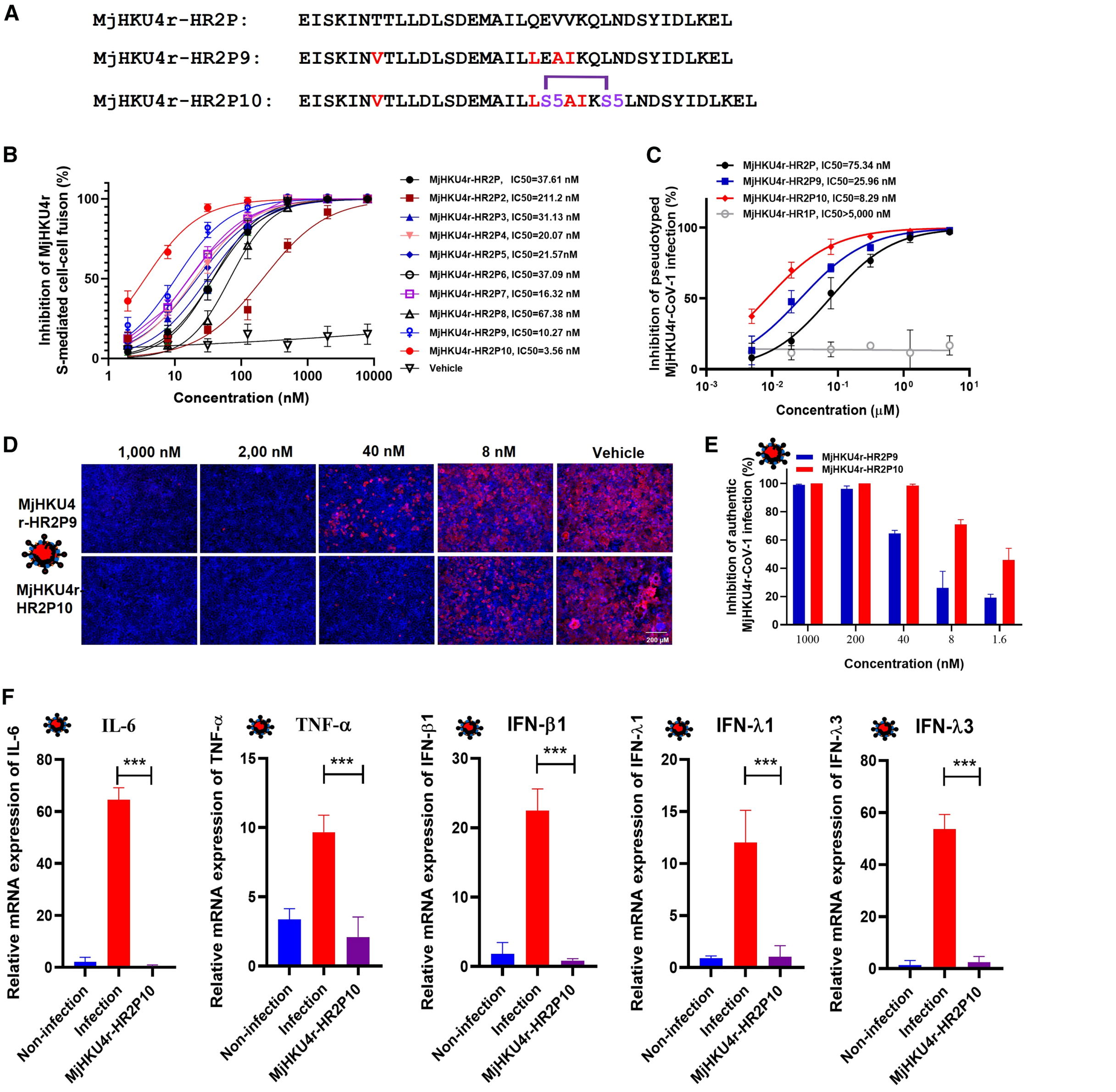

Figure 6. Antiviral potency of MjHKU4r-HR2P10 against MjHKU4r-CoV-1

(A) The sequences of MjHKU4r-HR2P9 and MjHKU4r-HR2P10; S5 indicates (S)-2-(4-pentenyl) alanine, which reacts to form the all-hydrocarbon staple.

(B and C) Inhibitory activity of MjHKU4r-HR2-derived peptides against MjHKU4r-S-mediated cell-cell fusion (B) or pseudovirus infection (C).

(D and E) Inhibitory efficacies of MjHKU4r-HR2P9 and MjHKU4r-HR2P10 against authentic MjHKU4r-CoV-1 infection, as measured by immunofluorescence assay with an antibody against the HKU4 N protein (right, scale bars, 200 μm). Red, NP; blue, nuclei (D) or RT-qPCR (E).

(F) MjHKU4r-HR2P10 at 40 nM completely blocked the upregulation of inflammatory genes (IL-6, TNF-α, IFN-β1, IFN-λ1, and IFN-λ3) induced by MjHKU4r-CoV infection in Caco-2 cells. *p* value is from a two-tailed unpaired t test (****p* < 0.001).

Data are represented as mean ± SEM of triplicate samples from a representative experiment of at least two independent experiments.

health. However, the specific pathogenic characteristics facilitated by its S protein remain unclear. Notably, our findings reveal that MjHKU4r-CoV-1's S protein mediates potent fusogenicity, suggesting that its potential pathogenicity in humans could be considerably higher than that of the previously reported MERS-related CoV, HKU4. To counter this, we crystallized the structure of its 6-HB fusion machine and revealed the MjHKU4r-CoV-1-specific HR1 target site. Thus, we were able to develop a series of HR2-derived fusion inhibitors capable of significantly blocking both MjHKU4r-CoV-1 S-mediated cell-cell fusion and infection by pseudotyped or authentic MjHKU4r-CoV-1.

CoVs primarily infect target cells through surface fusion or endosomal membrane fusion.[29,30] Either way, the fusogenic capacity of S protein critically influences viral infectivity and pathogenicity.[14,15] Particularly, the S protein on the infected cellular surface could directly drive cellular fusion between infected

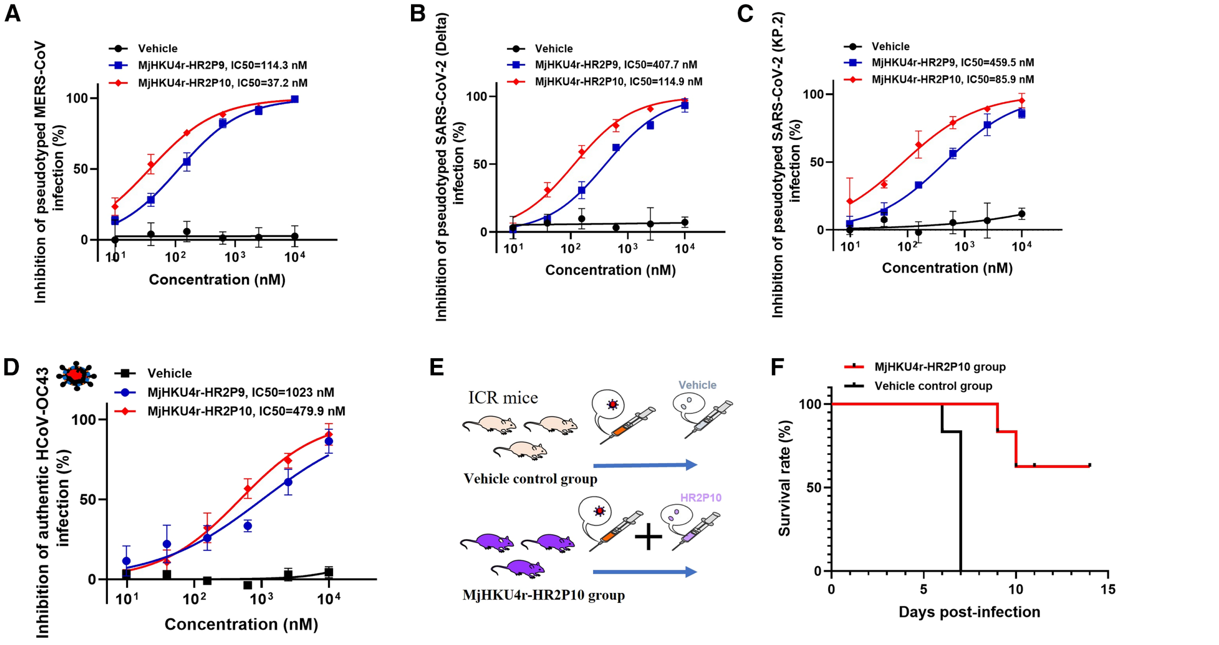

Figure 7. Antiviral potency and broad-spectrum efficacy of MjHKU4r-HR2P10 against multiple β-HCoVs

(A–C) Antiviral potency and broad-spectrum efficacy of MjHKU4r-HR2P9 and MjHKU4r-HR2P10 against MERS-CoV (A), SARS-CoV-2-Delta (B), and SARS-CoV-2-KP.2 (C).

(D–F) Antiviral potency of MjHKU4r-HR2P10 (D) and the protective efficacy *in vivo* of MjHKU4r-HR2P10 in the HCoV-OC43-infected mouse model, $n = 6$ (E and F). Data are represented as mean ± SEM of triplicate samples from a representative experiment of at least two independent experiments.

and adjacent uninfected cells, leading to syncytium formation *in vivo*.[31] These virus-mediated syncytia disrupt cellular function and life cycle and induce abnormal inflammatory responses and inappropriate type I IFN response.[19,32] Moreover, fusogenicity of the S protein enables cell-to-cell transmission of the virus, helping it evade humoral immunity or neutralizing antibody therapies.[24] Therefore, it is imperative to examine the specific fusogenicity feature of MjHKU4r-CoV-1 in order to elucidate its infectivity and pathogenicity and to aid in the development of effective antiviral treatments. In the current study, we established a cell-cell fusion model for MjHKU4r-CoV-1 without the assistance of exogenous proteases. This model differs from those of HKU4 or SARS-CoV by indicating a preference for the surface fusion route in MjHKU4r-CoV-1. The utilization of this cell-cell fusion model holds significant value in investigating viral entry mechanisms and conducting thorough evaluations of antiviral agents.[29] By using this fusion model, we identified MjHKU4r-CoV-1 as the MERS-related CoV to exhibit a fusion capacity surpassing even that of the current SARS-CoV-2. Notably, we observed that MjHKU4r-S-mediated cell-cell fusion triggered a substantial inflammatory response, strongly suggesting that MjHKU4r-CoV-1 could possess high *in vivo* pathogenic potential with corresponding high risk to human health. Intriguingly, MjHKU4r-CoV-1's S protein exhibits a more robust fusion capacity in human intestine-derived cells than that in lung-derived cells, hinting at a potential fecal-oral transmission route and the possibility of severe gastrointestinal symptoms, a hypothesis, however, that requires further investigation outside the scope of the present work.

We here characterized the crystal structure of MjHKU4r-CoV-1 6-HB, which showed potentially more stable interaction between HR1 and HR2 domains than that of SARS-CoV-2, thereby providing a clear understanding of the underlying mechanism driving the potent fusogenicity mediated by MjHKU4r-S. Additionally, this 6-HB structure can now serve as a reliable guide for the development of effective antiviral agents across different CoV strains. Antiviral peptides are a significant category of antiviral agents, as exemplified by the anti-HIV peptide T20 (enfuvirtide), the first fusion inhibitor approved for treating HIV infection.[33] Our previous work involved the successful development of MERS-HR2P, a fusion inhibitor derived from the MERS-CoV HR2 region. However, MERS-HR2P exhibited limited efficacy against MERS-CoV, only demonstrating activity at the micromolar level.[4] In contrast, peptides derived from MjHKU4r-HR2, particularly MjHKU4r-HR2P10, demonstrated exceptional efficacy in inhibiting MjHKU4r-S-mediated cell-cell fusion, PsV infection, and authentic MjHKU4r infection with IC_{50} values in the low nanomolar range. Moreover, MjHKU4r-HR2P10 significantly prevented infections by current SARS-CoV-2 variants, MERS-CoV and HCoV-OC43.

Based on prior structural and functional studies,[4,25] HR2-derived peptide (MjHKU4r-HR2P10) binds specifically to the viral HR1 domain through complementary hydrophobic and electrostatic interactions. These interactions are mediated by conserved residues at the "*a*" and "*d*" positions within the HR2 helix wheel, which align with corresponding "*g*" and "*e*" positions in the HR1 helix wheel, stabilizing the 6-HB critical for viral fusion.[4,25] Importantly, the "*g*" and "*e*" residues in HR1 helix wheel are highly

conserved across divergent CoVs, including MjHKU4r-CoV-1, MERS-CoV, SARS-CoV-2, and HCoV-OC43 (Figure S4). This evolutionary conservation of HR1's interaction interface likely underpins the observed broad-spectrum inhibitory activity of MjHKU4r-HR2P10 against these CoVs, as demonstrated in prior *in vitro* and *ex vivo* assays.

Comparative analysis of HR1 trimer interfaces reveals distinct hydrophobicity patterns. While MjHKU4r-CoV-1 and MERS-CoV retain phenylalanine (F1017/F1012) at this position, SARS-CoV-2 substitutes leucine (L938) and introduces a polar serine (S937) at the adjacent site. This combination reduces local hydrophobicity by ~50% (Eisenberg scale), potentially altering interhelical packing dynamics (Figures 4B and S5B). Nevertheless, compensatory interactions elsewhere in the 6-HB may mitigate these effects, underscoring the complexity of stability determinants in viral fusion machinery.

These findings highlight the potential of MjHKU4r-HR2P10 as a broadly effective fusion inhibitor for combating multiple circulating HCoVs and mitigating future MjHKU4r-CoV-1-related outbreaks. In addition, the resolved structure of MjHKU4r-6-HB provides critical insights for the development of other fusion inhibitors, including small-molecule compounds, peptides, or biomacromolecular agents. In conclusion, our comprehensive investigation into the fusogenicity, fusion core machinery, and fusion inhibitors of MjHKU4r-CoV-1 lays a solid foundation for the development of antiviral candidates able to prevent future MjHKU4r-CoV-1 epidemics.

Limitations of the study

First, the cell-cell fusion assays and pseudotyped or authentic MjHKU4r-CoV-1 infection studies were conducted *in vitro*. While these systems provide valuable insights, they may not fully replicate the complex physiological conditions observed during human infection. In particular, the gastrointestinal tropism of the virus in humans remains to be explored. Second, although MjHKU4r-HR2P10 exhibited significant protective efficacy in the lethal HCoV-OC43 mouse model, its *in vivo* activity against other CoVs has not yet been validated. Further studies utilizing additional animal models will be essential to address these limitations.

RESOURCE AVAILABILITY

Lead contact

For further information and access to viruses, peptides, or reagents used in the study, please address the lead contact, Lu Lu (lul@fudan.edu.cn).

Materials availability

Materials used in this study are available from the lead contact upon request.

Data and code availability

- The atomic coordinates and structure factors for the crystallographic structure have been deposited to the Protein DataBank (RCSB PDB: 8X5O).
- The RNA-seq data have been deposited at GEO (GSE296228). We do not report any custom computer code in this paper.
- Any additional information required to reanalyze the data reported in this work paper is available from the lead contact upon request.

ACKNOWLEDGMENTS

This work was supported by grants from the National Key R&D Program of China (2022YFC2604102 to L.L. and 2024YFA1307402 to Y.Z.), National Natural Science Foundation of China (grant nos. 92169112 to S.J., 82425033 and 82341036 to L.L., 82372221 to S.X., and 32471244 to Y.Z.), Major Project of Guangzhou National Laboratory (GZNL2023A01008 to L.L.), Shanghai Municipal Science and Technology Major Project (ZD2021CY001 to S.J., L.L., and S. X.), Shanghai Rising-Star Program (23QA1408700 to S.X.), and Development Fund for Shanghai Talents (S.X.).

AUTHOR CONTRIBUTIONS

L.L., S.J., P.Z., Y.Z., and S.X. conceived, planned, and supervised the experiments; S.X., Y.Z., J.C., L.W., F.J., T.L., W.X., X.W., and Q.W. performed the experiments and analyzed the data; S.X., J.C., and F.J. wrote the draft, while L.L., S.J., P.Z., Y.Z., and F.S. revised the manuscript.

DECLARATION OF INTERESTS

S.X., F.J., S.J., and L.L. are inventors in a patent application related to the pan-MERSr-CoV fusion inhibitors in this study.

STAR★METHODS

Detailed methods are provided in the online version of this paper and include the following:

- KEY RESOURCES TABLE
- EXPERIMENTAL MODEL AND STUDY PARTICIPANT DETAILS
  - Cell lines
  - Viruses
  - Mouse animal models
- METHOD DETAILS
  - Peptides
  - Plasmids
  - Immunization of mice with SARS-CoV-2-S1 or MERS-CoV-S1
  - Assays for S-mediated cell–cell fusion and its inhibition by peptides
  - RNA-seq sample preparation
  - RNA-seq data analysis
  - RNA isolation and RT-qPCR analysis
  - Inhibition of pseudotyped CoV infection
  - Circular dichroism spectroscopy and Tm value evaluation
  - Native polyacrylamide gel electrophoresis (N-PAGE)
  - Antiviral assays
  - Mouse infection studies
  - Expression and purification of fusion protein HR1-L6-HR2
  - Crystal structure determination
- QUANTIFICATION AND STATISTICAL ANALYSIS

SUPPLEMENTAL INFORMATION

Supplemental information can be found online at https://doi.org/10.1016/j.xcrm.2025.102277.

Received: February 20, 2025
Revised: April 7, 2025
Accepted: July 10, 2025
Published: August 6, 2025

REFERENCES

1. Cui, J., Li, F., and Shi, Z.-L. (2019). Origin and evolution of pathogenic coronaviruses. Nat. Rev. Microbiol. *17*, 181–192. https://doi.org/10.1038/s41579-018-0118-9.

2. Al-Tawfiq, J.A., Zumla, A., Gautret, P., Gray, G.C., Hui, D.S., Al-Rabeeah, A.A., and Memish, Z.A. (2014). Surveillance for emerging respiratory viruses. Lancet Infect. Dis. *14*, 992–1000. https://doi.org/10.1016/S1473-3099(14)70840-0.

3. Lu, G., Hu, Y., Wang, Q., Qi, J., Gao, F., Li, Y., Zhang, Y., Zhang, W., Yuan, Y., Bao, J., et al. (2013). Molecular basis of binding between novel human coronavirus MERS-CoV and its receptor CD26. Nature *500*, 227–231. https://doi.org/10.1038/nature12328.

4. Lu, L., Liu, Q., Zhu, Y., Chan, K.-H., Qin, L., Li, Y., Wang, Q., Chan, J.F.-W., Du, L., Yu, F., et al. (2014). Structure-based discovery of Middle East respiratory syndrome coronavirus fusion inhibitor. Nat. Commun. *5*, 3067. https://doi.org/10.1038/ncomms4067.

5. Zumla, A., Hui, D.S., and Perlman, S. (2015). Middle East respiratory syndrome. Lancet *386*, 995–1007. https://doi.org/10.1016/S0140-6736(15)60454-8.

6. Karani, A., Ombok, C., Situma, S., Breiman, R., Mureithi, M., Jaoko, W., Njenga, M.K., and Ngere, I. (2025). Low-Level Zoonotic Transmission of Clade C MERS-CoV in Africa: Insights from Scoping Review and Cohort Studies in Hospital and Community Settings. Viruses *17*, 125. https://doi.org/10.3390/v17010125.

7. Wang, Q., Qi, J., Yuan, Y., Xuan, Y., Han, P., Wan, Y., Ji, W., Li, Y., Wu, Y., Wang, J., et al. (2014). Bat origins of MERS-CoV supported by bat coronavirus HKU4 usage of human receptor CD26. Cell Host Microbe *16*, 328–337. https://doi.org/10.1016/j.chom.2014.08.009.

8. Yang, Y., Du, L., Liu, C., Wang, L., Ma, C., Tang, J., Baric, R.S., Jiang, S., and Li, F. (2014). Receptor usage and cell entry of bat coronavirus HKU4 provide insight into bat-to-human transmission of MERS coronavirus. Proc. Natl. Acad. Sci. USA *111*, 12516–12521. https://doi.org/10.1073/pnas.1405889111.

9. Ma, C.-B., Liu, C., Park, Y.-J., Tang, J., Chen, J., Xiong, Q., Lee, J., Stewart, C., Asarnow, D., Brown, J., et al. (2025). Multiple independent acquisitions of ACE2 usage in MERS-related coronaviruses. Cell *188*, 1693–1710.e18. https://doi.org/10.1016/j.cell.2024.12.031.

10. Park, Y.-J., Liu, C., Lee, J., Brown, J.T., Ma, C.-B., Liu, P., Gen, R., Xiong, Q., Zepeda, S.K., Stewart, C., et al. (2025). Molecular basis of convergent evolution of ACE2 receptor utilization among HKU5 coronaviruses. Cell *188*, 1711–1728.e21. https://doi.org/10.1016/j.cell.2024.12.032.

11. Jiang, S., and Wu, F. (2025). Global surveillance and countermeasures for ACE2-using MERS-related coronaviruses with spillover risk. Cell *188*, 1465–1468. https://doi.org/10.1016/j.cell.2025.02.004.

12. Chen, J., Yang, X., Si, H., Gong, Q., Que, T., Li, J., Li, Y., Wu, C., Zhang, W., Chen, Y., et al. (2023). A bat MERS-like coronavirus circulates in pangolins and utilizes human DPP4 and host proteases for cell entry. Cell *186*, 850–863.e16. https://doi.org/10.1016/j.cell.2023.01.019.

13. Li, F., Li, W., Farzan, M., and Harrison, S.C. (2005). Structure of SARS coronavirus spike receptor-binding domain complexed with receptor. Science *309*, 1864–1868. https://doi.org/10.1126/science.1116480.

14. Saito, A., Irie, T., Suzuki, R., Maemura, T., Nasser, H., Uriu, K., Kosugi, Y., Shirakawa, K., Sadamasu, K., Kimura, I., et al. (2022). Enhanced fusogenicity and pathogenicity of SARS-CoV-2 Delta P681R mutation. Nature *602*, 300–306. https://doi.org/10.1038/s41586-021-04266-9.

15. Suzuki, R., Yamasoba, D., Kimura, I., Wang, L., Kishimoto, M., Ito, J., Morioka, Y., Nao, N., Nasser, H., Uriu, K., et al. (2022). Attenuated fusogenicity and pathogenicity of SARS-CoV-2 Omicron variant. Nature *603*, 700–705. https://doi.org/10.1038/s41586-022-04462-1.

16. Bussani, R., Schneider, E., Zentilin, L., Collesi, C., Ali, H., Braga, L., Volpe, M.C., Colliva, A., Zanconati, F., Berlot, G., et al. (2020). Persistence of viral RNA, pneumocyte syncytia and thrombosis are hallmarks of advanced COVID-19 pathology. EBioMedicine *61*, 103104. https://doi.org/10.1016/j.ebiom.2020.103104.

17. Rajah, M.M., Hubert, M., Bishop, E., Saunders, N., Robinot, R., Grzelak, L., Planas, D., Dufloo, J., Gellenoncourt, S., Bongers, A., et al. (2021). SARS-CoV-2 Alpha, Beta, and Delta variants display enhanced Spike-mediated syncytia formation. EMBO J. *40*, e108944. https://doi.org/10.15252/embj.2021108944.

18. Zhou, Z., Zhang, X., Lei, X., Xiao, X., Jiao, T., Ma, R., Dong, X., Jiang, Q., Wang, W., Shi, Y., et al. (2021). Sensing of cytoplasmic chromatin by cGAS activates innate immune response in SARS-CoV-2 infection. Signal Transduct. Target. Ther. *6*, 382. https://doi.org/10.1038/s41392-021-00800-3.

19. Liu, X., Wei, L., Xu, F., Zhao, F., Huang, Y., Fan, Z., Mei, S., Hu, Y., Zhai, L., Guo, J., et al. (2022). SARS-CoV-2 spike protein-induced cell fusion activates the cGAS-STING pathway and the interferon response. Sci. Signal. *15*, eabg8744. https://doi.org/10.1126/scisignal.abg8744.

20. Li, S., Zhang, Y., Guan, Z., Li, H., Ye, M., Chen, X., Shen, J., Zhou, Y., Shi, Z.-L., Zhou, P., and Peng, K. (2020). SARS-CoV-2 triggers inflammatory responses and cell death through caspase-8 activation. Signal Transduct. Target. Ther. *5*, 235. https://doi.org/10.1038/s41392-020-00334-0.

21. Sethumadhavan, D.V., Jabeena, C.A., Govindaraju, G., Soman, A., and Rajavelu, A. (2021). The severity of SARS-CoV-2 infection is dictated by host factors? Epigenetic perspectives. Curr. Res. Microb. Sci. *2*, 100079. https://doi.org/10.1016/j.crmicr.2021.100079.

22. Wang, B., Xia, H., Peng, B.-H., Choi, E.-J., Tian, B., Xie, X., Makino, S., Bao, X., Shi, P.-Y., Menachery, V., and Wang, T. (2025). Pellino-1, a therapeutic target for control of SARS-CoV-2 infection and disease severity. Antiviral Res. *233*, 106059. https://doi.org/10.1016/j.antiviral.2024.106059.

23. Zeng, C., Evans, J.P., King, T., Zheng, Y.-M., Oltz, E.M., Whelan, S.P.J., Saif, L.J., Peeples, M.E., and Liu, S.-L. (2022). SARS-CoV-2 spreads through cell-to-cell transmission. Proc. Natl. Acad. Sci. USA *119*, e2111400119. https://doi.org/10.1073/pnas.2111400119.

24. Yu, S., Zheng, X., Zhou, Y., Gao, Y., Zhou, B., Zhao, Y., Li, T., Li, Y., Mou, J., Cui, X., et al. (2023). Antibody-mediated spike activation promotes cell-cell transmission of SARS-CoV-2. PLoS Pathog. *19*, e1011789. https://doi.org/10.1371/journal.ppat.1011789.

25. Xia, S., Liu, M., Wang, C., Xu, W., Lan, Q., Feng, S., Qi, F., Bao, L., Du, L., Liu, S., et al. (2020). Inhibition of SARS-CoV-2 (previously 2019-nCoV) infection by a highly potent pan-coronavirus fusion inhibitor targeting its spike protein that harbors a high capacity to mediate membrane fusion. Cell Res. *30*, 343–355. https://doi.org/10.1038/s41422-020-0305-x.

26. Yuan, Y., Cao, D., Zhang, Y., Ma, J., Qi, J., Wang, Q., Lu, G., Wu, Y., Yan, J., Shi, Y., et al. (2017). Cryo-EM structures of MERS-CoV and SARS-CoV spike glycoproteins reveal the dynamic receptor binding domains. Nat. Commun. *8*, 15092. https://doi.org/10.1038/ncomms15092.

27. Xia, S., Zhu, Y., Liu, M., Lan, Q., Xu, W., Wu, Y., Ying, T., Liu, S., Shi, Z., Jiang, S., and Lu, L. (2020). Fusion mechanism of 2019-nCoV and fusion inhibitors targeting HR1 domain in spike protein. Cell. Mol. Immunol. *17*, 765–767. https://doi.org/10.1038/s41423-020-0374-2.

28. Wang, C., Xia, S., Zhang, P., Zhang, T., Wang, W., Tian, Y., Meng, G., Jiang, S., and Liu, K. (2018). Discovery of hydrocarbon-stapled short α-helical peptides as promising Middle East respiratory syndrome coronavirus (MERS-CoV) fusion inhibitors. J. Med. Chem. *61*, 2018–2026. https://doi.org/10.1021/acs.jmedchem.7b01732.

29. Xia, S., Yan, L., Xu, W., Agrawal, A.S., Algaissi, A., Tseng, C.-T.K., Wang, Q., Du, L., Tan, W., Wilson, I.A., et al. (2019). A pan-coronavirus fusion inhibitor targeting the HR1 domain of human coronavirus spike. Sci. Adv. *5*, eaav4580. https://doi.org/10.1126/sciadv.aav4580.

30. Willett, B.J., Grove, J., MacLean, O.A., Wilkie, C., De Lorenzo, G., Furnon, W., Cantoni, D., Scott, S., Logan, N., Ashraf, S., et al. (2022). SARS-CoV-2 Omicron is an immune escape variant with an altered cell entry pathway. Nat. Microbiol. *7*, 1161–1179. https://doi.org/10.1038/s41564-022-01143-7.

31. Asarnow, D., Wang, B., Lee, W.-H., Hu, Y., Huang, C.-W., Faust, B., Ng, P.M.L., Ngoh, E.Z.X., Bohn, M., Bulkley, D., et al. (2021). Structural insight into SARS-CoV-2 neutralizing antibodies and modulation of syncytia. Cell *184*, 3192–3204.e16. https://doi.org/10.1016/j.cell.2021.04.033.

32. Ren, H., Ma, C., Peng, H., Zhang, B., Zhou, L., Su, Y., Gao, X., and Huang, H. (2021). Micronucleus production, activation of DNA damage response

CellPress OPEN ACCESS

and cGAS-STING signaling in syncytia induced by SARS-CoV-2 infection. Biol. Direct *16*, 20. https://doi.org/10.1186/s13062-021-00305-7.

33. Kilby, J.M., and Eron, J.J. (2003). Novel therapies based on mechanisms of HIV-1 cell entry. N. Engl. J. Med. *348*, 2228–2238. https://doi.org/10.1056/NEJMra022812.

34. Xia, S., Wang, L., Jiao, F., Yu, X., Xu, W., Huang, Z., Li, X., Wang, Q., Zhu, Y., Man, Q., et al. (2023). SARS-CoV-2 Omicron subvariants exhibit distinct fusogenicity, but similar sensitivity, to pan-CoV fusion inhibitors. Emerg. Microbes Infect. *12*, 2178241. https://doi.org/10.1080/22221751.2023.2178241.

35. Xia, S., Lan, Q., Zhu, Y., Wang, C., Xu, W., Li, Y., Wang, L., Jiao, F., Zhou, J., Hua, C., et al. (2021). Structural and functional basis for pan-CoV fusion inhibitors against SARS-CoV-2 and its variants with preclinical evaluation. Signal Transduct. Target. Ther. *6*, 288. https://doi.org/10.1038/s41392-021-00712-2.

36. Lan, Q., Wang, L., Jiao, F., Lu, L., Xia, S., and Jiang, S. (2023). Pan-coronavirus fusion inhibitors to combat COVID-19 and other emerging coronavirus infectious diseases. J. Med. Virol. *95*, e28143. https://doi.org/10.1002/jmv.28143.

37. Su, X., Huang, Z., Xu, W., Wang, Q., Xing, L., Lu, L., Jiang, S., and Xia, S. (2023). IgG Fc-binding peptide-conjugated Pan-CoV fusion inhibitor exhibits extended in vivo half-life and synergistic antiviral effect when combined with neutralizing antibodies. Biomolecules *13*, 1283. https://doi.org/10.3390/biom13091283.

38. Yang, Y., Liu, C., Du, L., Jiang, S., Shi, Z., Baric, R.S., and Li, F. (2015). Two mutations were critical for bat-to-human transmission of Middle East respiratory syndrome coronavirus. J. Virol. *89*, 9119–9123. https://doi.org/10.1128/JVI.01279-15.

39. Xue, S., Wang, X., Wang, L., Xu, W., Xia, S., Sun, L., Wang, S., Shen, N., Yang, Z., Huang, B., et al. (2022). A novel cyclic γ-AApeptide-based long-acting pan-coronavirus fusion inhibitor with potential oral bioavailability by targeting two sites in spike protein. Cell Discov. *8*, 88. https://doi.org/10.1038/s41421-022-00455-6.

40. Chen, Y.H., Yang, J.T., and Chau, K.H. (1974). Determination of the helix and beta form of proteins in aqueous solution by circular dichroism. Biochemistry *13*, 3350–3359. https://doi.org/10.1021/bi00713a027.

41. Kabsch, W. (2010). XDS. Acta Crystallogr. D Biol. Crystallogr. *66*, 125–132. https://doi.org/10.1107/S0907444909047337.

42. McCoy, A.J., Grosse-Kunstleve, R.W., Adams, P.D., Winn, M.D., Storoni, L.C., and Read, R.J. (2007). Phaser crystallographic software. J. Appl. Crystallogr. *40*, 658–674. https://doi.org/10.1107/S0021889807021206.

43. Emsley, P., and Cowtan, K. (2004). Coot: model-building tools for molecular graphics. Acta Crystallogr. D Biol. Crystallogr. *60*, 2126–2132. https://doi.org/10.1107/S0907444904019158.

44. Afonine, P.V., Poon, B.K., Read, R.J., Sobolev, O.V., Terwilliger, T.C., Urzhumtsev, A., and Adams, P.D. (2018). Real-space refinement in PHENIX for cryo-EM and crystallography. Acta Crystallogr. D Struct. Biol. *74*, 531–544. https://doi.org/10.1107/S2059798318006551.

STAR★METHODS

KEY RESOURCES TABLE

REAGENT or RESOURCE	SOURCE	IDENTIFIER
**Antibodies**		
Rabbit anti-Tylonycteris batcoronavirus HKU4 N Protein polyclonal antibody	Chen et al.[12]	N/A
Goat Anti-Rabbit IgG H&L (Cy3) preadsorbed	Abcam	Cat#: ab6939; RRID:AB_955021
**Bacterial and virus strains**		
HCoV-OC43	ATCC	VR-1558
MjHKU4r-CoV-1	Chen et al.[12]	N/A
**Chemicals, peptides, and recombinant proteins**		
MjHKU4r-HR1P, Table S1	This study	N/A
MjHKU4r-HR2P, Table S1	This study	N/A
MjHKU4r-HR2P2, Table S1	This study	N/A
MjHKU4r-HR2P3, Table S1	This study	N/A
MjHKU4r-HR2P4, Table S1	This study	N/A
MjHKU4r-HR2P5, Table S1	This study	N/A
MjHKU4r-HR2P6, Table S1	This study	N/A
MjHKU4r-HR2P7, Table S1	This study	N/A
MjHKU4r-HR2P8, Table S1	This study	N/A
MjHKU4r-HR2P9, Table S1	This study	N/A
MjHKU4r-HR2P10, Table S1	This study	N/A
**Critical commercial assays**		
Luciferase Assay System	Promega	Cat# E1500
Cell Culture Lysis 5X Reagent	Promega	Cat# E1531
Vigofect	Vigorous	Cat# T001
One Step qRT-PCR SYBR Green Kit	Vazyme	Cat# Q221
**Deposited data**		
Crystal structure of the post-fusion core of MjHKUr-CoV-1 spike protein	This study	PDB ID: 8X5O
RNA-seq data of S-mediated cell fusion	This study	GEO: GSE296228
**Experimental models: Cell lines**		
293T	ATCC	Cat# CRL-3216
Caco-2	ATCC	Cat# HTB-37
Calu-3	ATCC	Cat# HTB-55
**Oligonucleotides**		
Primers for IFN-β1 (RT-qPCR), Table S3	This study	N/A
Primers for TNF-α (RT-qPCR), Table S3	This study	N/A
Primers for IL-6 (RT-qPCR), Table S3	This study	N/A
Primers for CCL5 (RT-qPCR), Table S3	This study	N/A
Primers for CXCL2 (RT-qPCR), Table S3	This study	N/A
Primers for IFN-λ1 (RT-qPCR), Table S3	This study	N/A
Primers for IFN-λ2 (RT-qPCR), Table S3	This study	N/A
Primers for IFN-λ3 (RT-qPCR), Table S3	This study	N/A
F-MjHKU4r-CoV-1-ORF5 CTTCGTGTTGATAAT GGTACTTCC	Chen et al.[12]	N/A

(*Continued on next page*)

Continued

REAGENT or RESOURCE	SOURCE	IDENTIFIER
R-MjHKU4r-CoV-1-ORF5 AGCAGAGTGCACATA GAAACA	Chen et al.[12]	N/A
**Recombinant DNA**		
pAAV-IRES-EGFP	Xia et al.[29]	N/A
pAAV-SARS-CoV-2-spike D614G-IRES-EGFP	Xia et al.[34]	N/A
pAAV-MERS-spike-IRES-EGFP	Xia et al.[29]	N/A
pAAV-SARS-spike-IRES-EGFP	Xia et al.[29]	N/A
pAAV-HKU4-spike-IRES-EGFP	This study	N/A
pAAV-MjHKU4r-spike-IRES-EGFP	This study	N/A
pNL4-3.Luc.R-E	Xia et al.[34]	N/A
pcDNA3.1	Xia et al.[34]	N/A
pcDNA3.1-SARS-CoV-2-BA.2.75-S	Xia et al.[34]	N/A
pcDNA3.1-SARS-CoV-2-XBB.1.16-S	Xia et al.[34]	N/A
pcDNA3.1-SARS-CoV-2-Delta-S	Xia et al.[34]	N/A
pcDNA3.1-SARS-CoV-2-KP.2-S	This study	N/A
pcDNA3.1-SARS-CoV-S	Xia et al.[29]	N/A
pcDNA3.1-MERS-CoV-S	Xia et al.[29]	N/A
pcDNA3.1-MjHKU4r -S	This study	N/A
**Software and algorithms**		
GraphPad Prism (version 8.0.2)	GraphPad Software	https://www.graphpad.com/
EMBOSS Needle	EMBOSS Tools	http://www.ebi.ac.uk/Tools/psa/emboss_needle/
BioEdit (v7.1.3.0)	Software.informer	http://www.mbio.ncsu.edu/BioEdit/bioedit.html
DESeq2 (v1.38.1)	Bioconductor	https://doi.org/10.18129/B9.bioc.DESeq2
GSEABase (v1.60.0)	Bioconductor	https://doi.org/10.18129/B9.bioc.GSEABase
enrichplot (v1.18.1)	Bioconductor	https://doi.org/10.18129/B9.bioc.enrichplot
Primer Premier 6	Premier Biosoft	https://www.premierbiosoft.com
pheatmap (v1.0.12)	CRAN	https://doi.org/10.32614/CRAN.package.pheatmap
**Other**		
Crystal structure of the post-fusion core of SARS-CoV-2 (PDB entry 6LXT)	Xia et al.[25]	https://www.rcsb.org/structure/6LXT
Crystal structure of the post-fusion core of MERS-CoV (PDB entry 4NJL)	Lu et al.[4]	https://www.rcsb.org/structure/4NJL

EXPERIMENTAL MODEL AND STUDY PARTICIPANT DETAILS

Cell lines

The 293T, Calu-3 and Caco-2 cell lines were obtained from the American Type Culture Collection (ATCC). All cell lines were cultured in Dulbecco's Modified Eagle's Medium (DMEM) supplemented with 100 U/ml penicillin, 100 μg/mL streptomycin, and 10% heat-inactivated fetal calf serum (FCS). These cell lines were authenticated by the supplier and were routinely tested for mycoplasma contamination.

Viruses

The HCoV-OC43 (VR-1558) strain was obtained from the ATCC. MjHKU4r-CoV-1 was isolated by the Wuhan Institute of Virology. All experimental procedures involving live MjHKU4r-CoV-1 were conducted in strict accordance with the approved standard operating protocols at the Biosafety Level 3 (BSL-3) facility of the Wuhan Institute of Virology.

Mouse animal models

Newborn ICR mice (3-day-old) bred from pregnant mice purchased from the Animal Center of Fudan University were used for infection studies. Six-week-old female Balb/c mice obtained from the same facility were used for immunization experiments. All animal procedures were approved by the Institutional Laboratory Animal Care Committee of Fudan University (approval number: 20240229-054).

METHOD DETAILS

Peptides

All peptides were custom-synthesized by Synpeptide Co., Ltd (http://www.synpeptide.com) with ≥95% purity, as verified by high-performance liquid chromatography (HPLC). Each peptide was modified with N-terminal acetylation and C-terminal amidation to enhance stability. Notably, MjHKU4r-HR2P10 incorporates two (S)-2-(4-pentenyl)alanine (S5) substitutions at positions 1278E and 1282Q, enabling the formation of an all-hydrocarbon staple through olefin metathesis, as previously described.[28]

Plasmids

Plasmids expressing the genes of MjHKU4r-S were a gift from Prof. Zhou. The genes encoding HKU4-S, MERS-CoV-S, SARS-CoV-2-S (Delta, BA.2.75, XBB.1.16, KP.2), SARS-CoV-S, and plasmids Pc-DNA3.1 and pAAV-IRES-EGFP, as well as the luciferase reporter vector (pNL4-3.Luc.R-E−), were maintained in our laboratory.[29]

Immunization of mice with SARS-CoV-2-S1 or MERS-CoV-S1

Briefly, SARS-CoV-2-S1 or MERS-CoV-S1 (5 μg) formulated with an equal volume of Imject Alum adjuvant was used to vaccinate Balb/c mice (six-week-old) three times at two-week intervals. At the 42nd day, sera were isolated from blood samples to inhibit S-mediated cell-cell fusion or PsV infection in 1:300 dilution.

Assays for S-mediated cell–cell fusion and its inhibition by peptides

As previously described,[29,35–37] briefly, Calu-3 cells or Caco-2 cells were used as target cells. 293T cells transfected with one of the S protein expression vectors, including 293T/MjHKU4r-S/GFP, 293T/HKU4-S/GFP, 293T/MERS-CoV-S/GFP, 293T/SARS-CoV-S/GFP, 293T/SARS-CoV-2-S (KP.2)/GFP or empty plasmid pAAV-IRES-EGFP, were used as effector cells. Effector cells and target cells were cocultured in DMEM for indicated time. After incubation, fused and unfused cells were counted under an inverted fluorescence microscope (Nikon Eclipse Ti-S).

The inhibitory activity of peptides against S-mediated cell–cell fusion was assessed as previously described.[34,38,39] Briefly, pre-24 h, 2×10^4 cells/well Caco-2 cells were seeded in a 96-well plate. Then, 10^4 cells/well effector cells (293T/S/GFP) with or without tested peptide at the indicated concentrations were added for a 4-h culture at 37°C. 293T/EGFP cells were used as a negative control. Fusion rate was calculated based on the number of the fused and unfused cells.[4]

RNA-seq sample preparation

Briefly, 293T cells were transfected with the pAAV-IRES-EGFP vector plasmid encoding HKU4, MjHKU4r-CoV-1, or SARS-CoV-2 (KP.2) S proteins to serve as effector cells. Caco-2 cells, which naturally express human ACE2 receptors on their membrane surface, were used as target cells. Effector cells (293T/S/GFP) were collected, resuspended, and subsequently co-incubated with the target cells (Caco-2) for 36 h at 37°C. Following incubation, cells were lysed using RNAiso Plus (Takara, Japan), and RNA extraction was performed according to the manufacturer's instructions. Purified RNA (10 μL) was used for cDNA synthesis and library preparation. RNA sequencing was conducted on the Illumina NovaSeq 6000 PE150 platform.

RNA-seq data analysis

Each group consisted of four biological replicates. After RNA extraction, RNA sequencing was performed using the Illumina NovaSeq 6000 PE150 platform (Illumina, USA). Raw read counts were generated by ApexBio Technology (USA). Differentially expressed genes (DEGs) were identified using DESeq2 (v1.38.1) based on log fold change (logFC) and adjusted p-values (P.adj). Gene set enrichment analysis (GSEA) was performed using hallmark (version: h.all.v2022.1.Hs.symbols.gmt) and Gene Ontology (GO) (version: c5.go.bp.v2023.1.Hs.symbols.gmt) gene sets via GSEABase (v1.60.0) with enrichment plots visualized using enrichplot (v1.18.1). Heatmaps of genes involved in related pathways were generated using pheatmap (v1.0.12) to illustrate relative expression levels.

RNA isolation and RT-qPCR analysis

Total RNA was isolated as described previously.[19] RT-qPCR was performed to quantify RNA levels of cytokines using the SYBR Green PCR kit. Primers (Table S3) for qPCR were designed using Primer Premier6 software.

Inhibition of pseudotyped CoV infection

To package pseudotyped coronavirus, 293T cells were co-transfected with pNL4–3.luc.RE (HIV-1 backbone expressing the luciferase reporter) and pcDNA3.1-MjHKU4r-S using VigoFect. After 48 h, the supernatant containing pseudotyped particles was

harvested at 72 h post-transfection, centrifuged at 3000 × g for 10 min, and stored in −80°C. To detect the inhibitory activity of peptides against MjHKU4r-CoV-1 PsV, Caco-2 cells were plated in wells of a 96-well plate (10^4 cells per well) one day prior to infection. MjHKU4r-CoV-1 PsV was mixed with an equal volume of a peptide in indicated concentration for coincubation at 37°C for 30 min. Then, the mixture was transferred to the Caco-2 cells. After 12 h, medium was refreshed for an additional 48 h culture. Finally, luciferase activity was analyzed by the Luciferase Assay System.

Circular dichroism spectroscopy and Tm value evaluation

The peptides (10 μM) or their mixtures in PBS were measured on a Jasco-815 circular dichroism spectrometer with scanning wavelength ranging between 198 and 260 nm. The $[\theta]_{222}$ value of −33 (10^{-3} deg cm^2 $dmol^{-1}$) was taken to correspond to 100% a-helical.[40] Thermal denaturation was detected at 222 nm with a 5°C/min thermal gradient detection.[4]

Native polyacrylamide gel electrophoresis (N-PAGE)

N-PAGE was conducted as described elsewhere. Briefly, each MjHKU4r-HR2P peptide (40 μM) in PBS was incubated with MjHKU4r-HR1P (20, 40, 80 μM), respectively, at 37°C for 1 h and then loaded on a tris-glycine gel (12%) with tricine glycine running buffer (pH 8.6). Finally, staining was performed with Coomassie blue, and the images were visualized on the FluorChem Imaging System.

Antiviral assays

As described previously,[12] target cells (Caco-2) were plated in wells of a 48-well plate and cultured overnight. Each peptide was serially diluted 5-fold and incubated with 100 PFU of MjHKU4r-CoV-1 strain[12] at 34°C for 30 min. The virus-peptide mixture was then transferred to Caco-2 cell monolayers in triplicate. After a 1-h incubation, the mixture was removed. Cells were washed with DMEM twice, and fresh DMEM containing 2% FBS and peptide in indicated concentrations was added. At 72 h post-infection, viral RNA was extracted from culture supernatants for the quantification of viral genomic copies by RT-qPCR. Cells were fixed with 4% paraformaldehyde at room temperature for 40 min, and IF staining was performed, as described previously.[12]

Mouse infection studies

Newborn mice (ICR, 3-day-old) were bred from pregnant mice purchased from the Animal Center of Fudan University. Newborn mice were intranasally administered MjHKU4r-HR2P10 peptide (3 mg/kg in 2 μL PBS, n = 6) or PBS (viral group, n = 6) 30 min after intranasal challenge with HCoV-OC43 at a viral dose of 10^2 $TCID_{50}$. Mouse survival rate and body weight variations were recorded up to 2 weeks after infection.

Expression and purification of fusion protein HR1-L6-HR2

The coding sequences of HR1 (residues 989–1,064) and HR2 (residues 1,253–1,296) domains of MjHKU4r S2 subunits were tandem linked though a 6-residue linker (L6: SGGRGG). The resulting sequences encoding the fused HR1-L6-HR2 protein were then cloned into a modified pET-32a vector containing a His6-TRX tag upstream of the multiple cloning sites. The recombinant plasmid was expressed in *Escherichia coli* BL21. Cells were grown in lysogeny broth (LB) media supplemented with 50 μg/mL ampicillin at 37°C after inducing with 1 mM IPTG for 12 h at 16°C overnight. Cells were harvested by centrifugation at 4500*g* for 10 min at 4°C. Then, cells were resuspended in buffer containing 25 mM Tris–HCl, pH8.0, and 200 mM NaCl and were lysed by high-pressure homogenizer. Fusion proteins were isolated by Ni-affinity chromatography, and the TRX tag was removed by TEV Protease (1:100 w/w) cleavage. HR1-L6-HR2 protein was concentrated and gel-filtered on a 10/300 Superdex 75 (GE Healthcare) column. Peak fractions containing HR1-L6-HR2 trimer were pooled and concentrated to 15 mg/mL through centrifugation.

Crystal structure determination

Crystals were obtained at 16°C for 5 days using the hanging drop vapor diffusion method by mixing equal volume of protein solution (HR1-HR2, 15 mg/mL) and reservoir solution (2.8M Sodium Acetate/HCl, pH 7.00, for HR1-HR2). Then, crystals were flash-frozen and transferred to liquid nitrogen for data collection. On the in-house (Institute of Biophysics, Chinese Academy of Sciences) X-ray source (MicroMax 007 generator (Rigaku, Japan)) combined with Varimax HR optics (Rigaku, Japan), crystals at 100 K were diffracted at a wavelength of 1.5418 Å. A native set of X-ray diffraction data was collected with the Satun944HG (Rigaku, Japan) with an exposure time of 15s per image and was indexed and processed using XDS.[41] The space group of the collected dataset was P 63 2 2 for the HR1-HR2 crystal. Molecular replacement was performed with PHENIX.phaser[42] to solve the phasing problem, using the crystal structure of SARS-CoV-2 HR1-HR2 (PDB entry 6LXT) as a search model. The final model was manually adjusted in COOT[43] and refined with Phenix.refine.[44] Data collection statistics and refinement statistics are given in Table S2. Coordinates were deposited in the RCSB Protein DataBank with PDB entry 8X5O for HR1-HR2.

QUANTIFICATION AND STATISTICAL ANALYSIS

Statistical analyses were carried out using GraphPad Prism 8.0.2. Data are represented as mean ± SEM. Analyses of independent data were performed by Student's unpaired two-tailed t test. p values less than 0.05 were considered significant; $^{*}p < 0.05$, $^{**}p < 0.01$, $^{***}p < 0.001$, and $^{****}p < 0.0001$.

Cell Reports Medicine, Volume *6*

Supplemental information

MERS-related coronavirus circulating in pangolins exhibits strong fusogenicity in human cells and high sensitivity to fusion inhibitors

Shuai Xia, Fanke Jiao, Jing Chen, Lijue Wang, Tianyu Lu, Qian Wang, Wei Xu, Xinling Wang, Fei Sun, Yun Zhu, Peng Zhou, Shibo Jiang, and Lu Lu

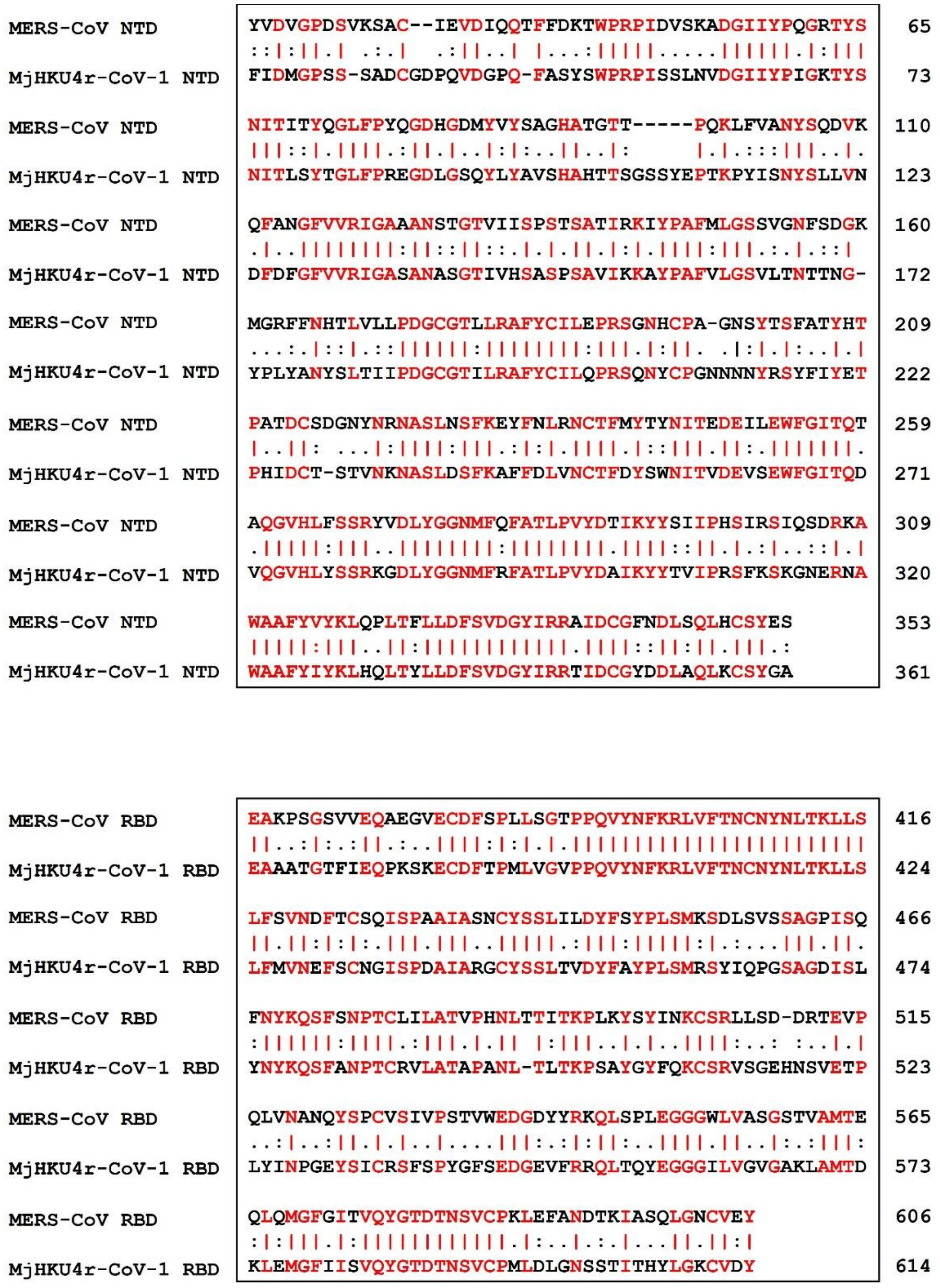

Figure S1. **Sequence similarities between the NTD/RBD domains in S1 of MjHKU4r-CoV-1 and those of MERS-CoV**. Identical amino acid residues are highlighted in red. Related to Figure 1A.

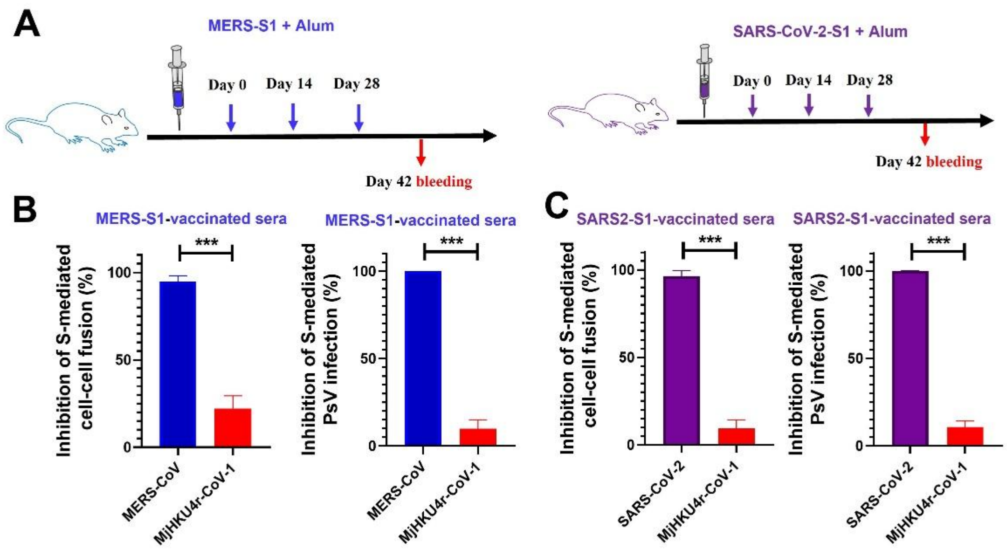

Figure S2. Efficacy of MERS-CoV- or SARS-CoV-2 S1-immunized mouse serum (at 1:300 dilution) against MjHKU4r-S-mediated cell-cell fusion or pseudovirus infection. Data are represented as mean ±SEM of triplicate samples from a representative experiment of at least two independent experiments. p value is from a two-tailed unpaired t-test (***$p<0.001$). Related to Figure 1.

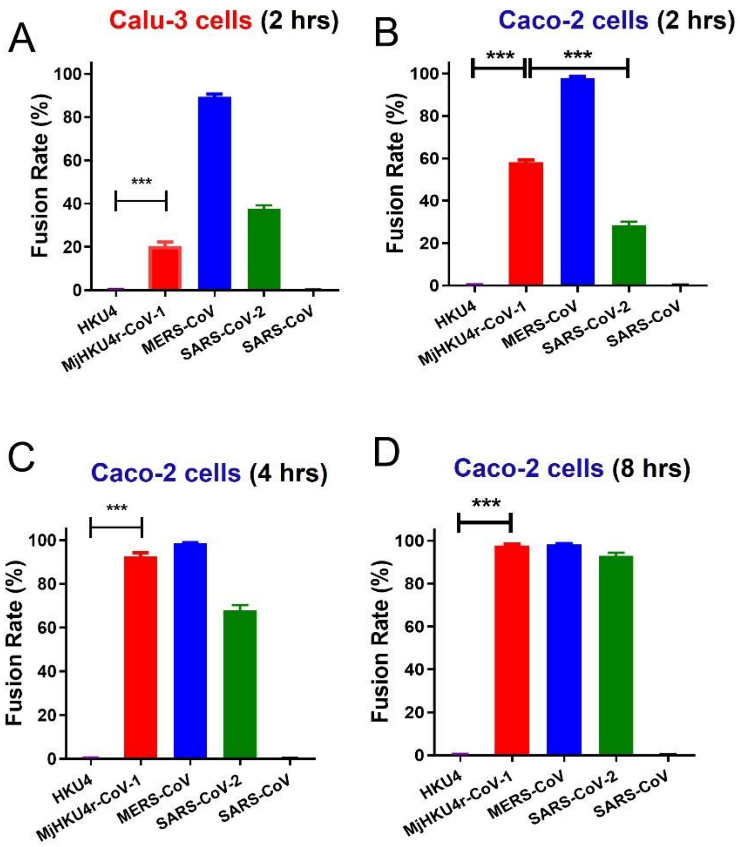

Figure S3. Fusion rates evaluation of MjHKU4-S on Caco-2 cells. Related to Figure 1.

(A-B) Fusion rates mediated by HKU4, MjHKU4-CoV-1, MERS-CoV, SARS-CoV-2 (KP.2) and SARS-CoV S proteins on Calu-3 cells (A) or on Caco-2 cells (B) after coculture for 2 hrs. **(C-D)** Fusion rates mediated by HKU4, MjHKU4-CoV-1, MERS-CoV, SARS-CoV-2 (KP.2) and SARS-CoV S protein on Caco-2 cells after coculture for 4 hrs (C) or 8 hrs (D). Data are represented as mean ±SEM of triplicate samples from a representative experiment of at least two independent experiments. p value is from a two-tailed unpaired t-test (***p<0.001). Related to Figure 1.

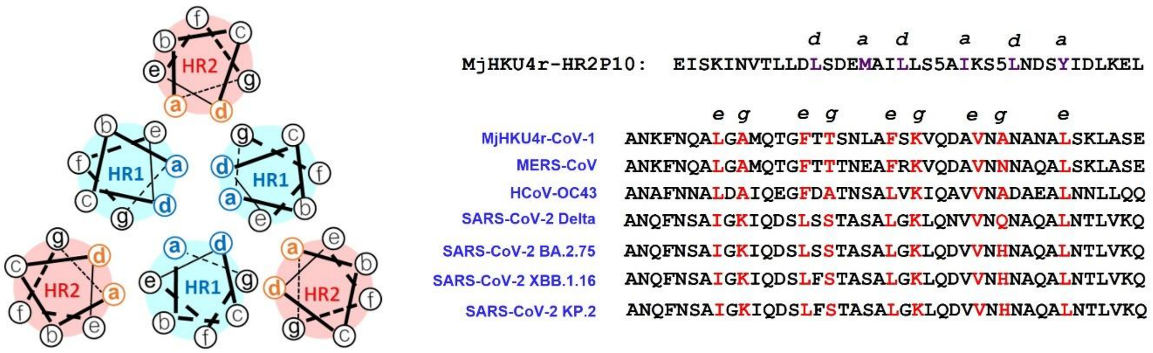

Figure S4. Illustration of 6-HB formation by the HR1 and HR2 domains in the S2 subunit of the MjHKU4r-CoV-1 S protein, along with the mechanism of MjHKU4r-HR2P10 targeting the viral HR1 domain. Related to Figures 3 and 6.

The HR1 trimer is formed by interactions between the amino acid residues (mostly hydrophobic) at the "*a*" and "*d*" positions in the helix wheel of adjacent HR1 domains. This trimer subsequently binds three HR2 domains (or three MjHKU4r-HR2P10 molecules) through interactions with the exposed hydrophobic grooves on the HR1 trimer. Specifically, this binding occurs via interactions between the hydrophobic amino acid residues at the "*a*" and "*d*" positions in the helix wheel of HR2 domains (or MjHKU4r-HR2P10) and those at the "*e*" and "*g*" positions in the helix wheel of HR1 domains.

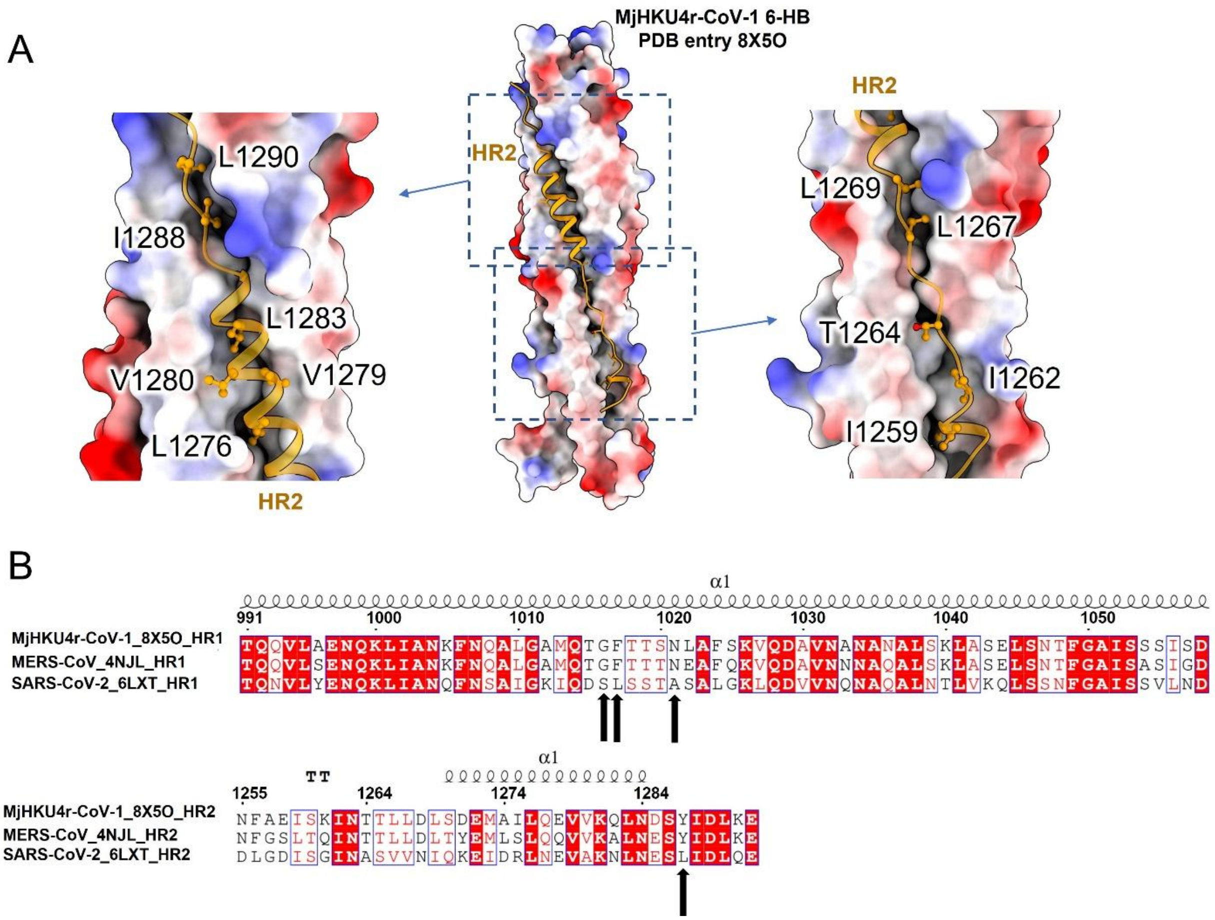

Figure S5. Structural and sequence details of the MjHKU4r 6-HB fusion machine. Related to Figure 4.

(A) The interactions between HR1 and HR2 domains within the hydrophobic grooves of HR1 are displayed in a zoomed-in view and labeled. The hydrophobic grooves of the HR1 trimer are shown as surface electrostatic potential. (B) Multiple sequence alignment of the conserved regions in the HR1 and HR2 domains of the S proteins of MjHKU4r-CoV-1, MERS-CoV, and SARS-CoV-2. Important residues are marked with black arrowheads.

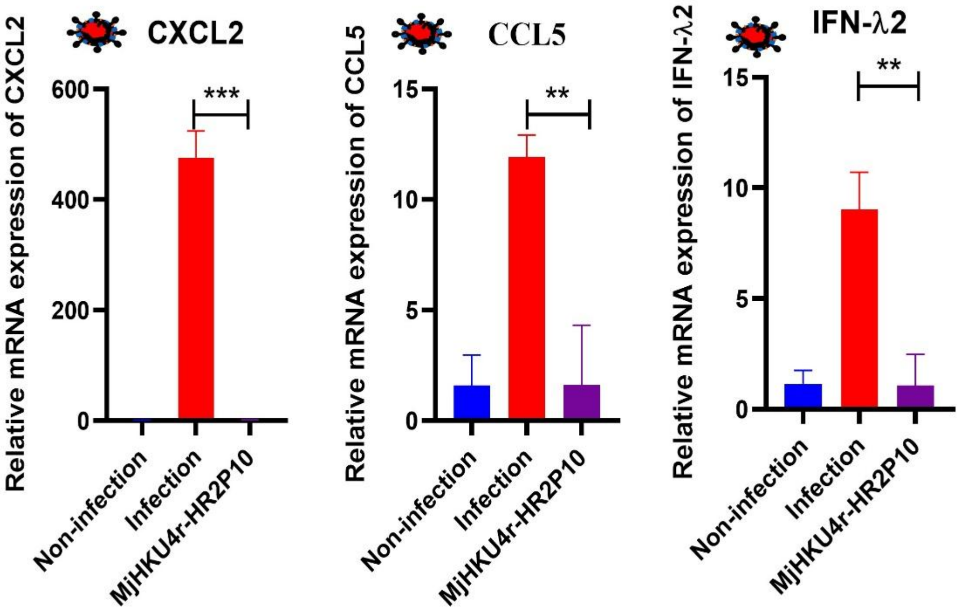

Figure S6. MjHKU4r-HR2P10 at 40 nM completely blocked the upregulation of inflammatory genes (CXCL2, CCL5, IFN-λ2) induced by MjHKU4r-CoV-1 infection in Caco-2 cells. Data are represented as mean ±SEM of triplicate samples from a representative experiment of at least two independent experiments. p value is from a two-tailed unpaired t-test (**p<0.01, ***p<0.001). Related to Figure 6.

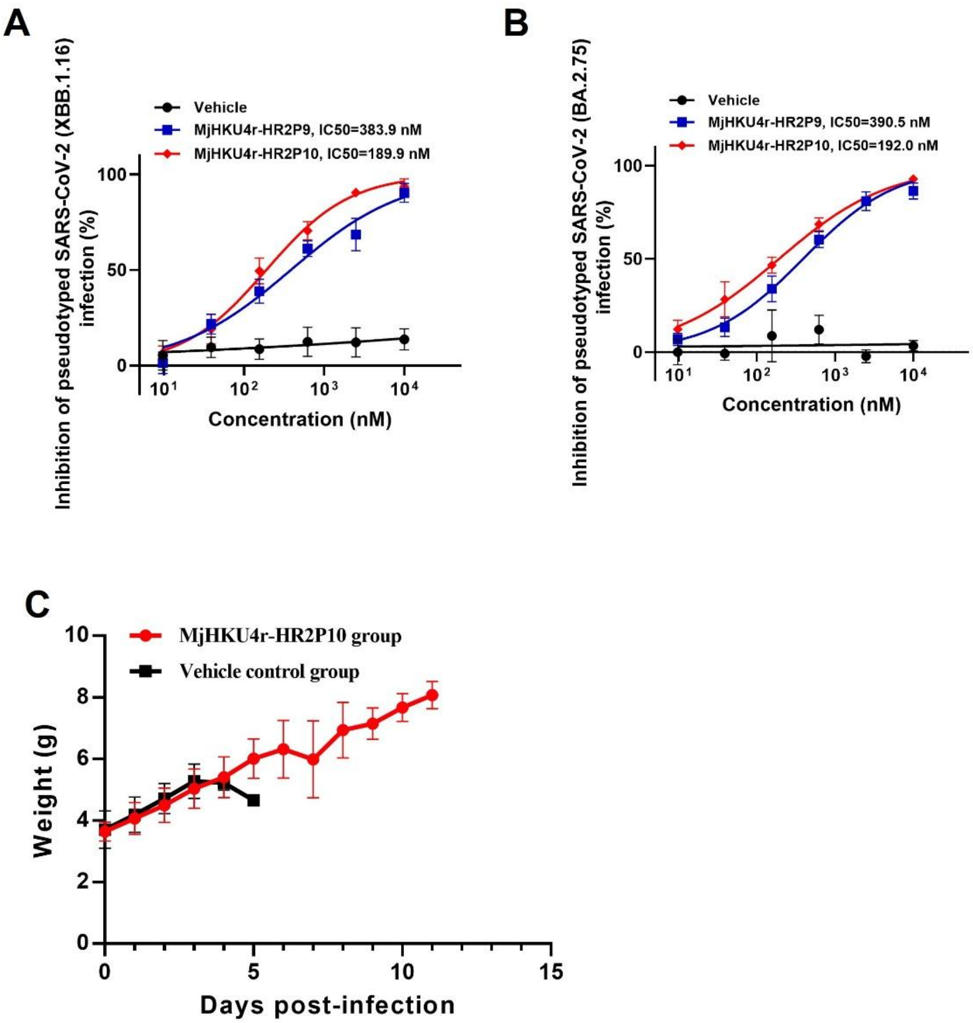

Figure S7. Inhibitory efficacy of MjHKU4r-HR2P10 against pseudotyped SARS-CoV-2-XBB.1.16 (A) and SARS-CoV-2-BA.2.75 (B) infections, and the body weight change of newborn mice treated with MjHKU4r-HR2P10 after HCoV-OC43 challenge (C). Data are represented as mean ±SEM. Related to Figure 7.

Table S1. The sequence of MjHKU4r-HR2P to MjHKU4r-HR2P10, related to Figure 6.

Peptides	Sequence
**MjHKU4r-HR2P**	EISKINTTLLDLSDEMAILQEVVKQLNDSYIDLKEL
**MjHKU4r-HR2P2**	PNFAEISKINTTLLDLSDEMAILQEVVKQLNDSYIDLKEL
**MjHKU4r-HR2P3**	EISKINTTLLDLSDEMAILQEVVKQLNDSLIDLKEL
**MjHKU4r-HR2P4**	EISKINTTLLDLSDEMAILQEAIKQLNDSYIDLKEL
**MjHKU4r-HR2P5**	EISKINVTLLDLSDEMAILQEVVKQLNDSYIDLKEL
**MjHKU4r-HR2P6**	EISKINVTFLDLSDEMAILQEVVKQLNDSYIDLKEL
**MjHKU4r-HR2P7**	EISKINTTLLDLSDEMAILLEVVKQLNDSYIDLKEL
**MjHKU4r-HR2P8**	EISKINTTLLDLSDFMAILLEVVKQLNDSYIDLKEL
**MjHKU4r-HR2P9**	EISKINVTLLDLSDEMAILLEAIKQLNDSYIDLKEL
**MjHKU4r-HR2P10**	EISKINVTLLDLSDEMAILL-S5-AIK-S5-LNDSYIDLKEL

Table S2. Data collection and refinement statistics, related to Figure 4.

	**MjHKU4r HR1-HR2 PDB entry 8X5O**
**Data collection**	
Space group	P 63 2 2
Cell dimensions	
a, b, c (Å)	198.7, 198.7, 64.1
α, β, γ (°)	90, 90, 120
Wavelength (Å)	1.5418
Resolution (Å)	34.42 -2.67 (2.80 - 2.67) †
R_{merge}	0.43 (2.11)
Mean I/σ(I)	12.5 (2.5)
Completeness (%)	99.5 (97.0)
Redundancy	37.6 (37.3)
**Refinement**	
Resolution (Å)	33.12 -2.67
No. of reflections	21595
Reflections in test set	1094
R_{work}/R_{free}	0.241/0.292
No. of protein atoms	
	2489
Water & Ligands	0
Root mean square (rms) deviations	
Bond lengths (Å)	0.003
Bond angles (°)	0.49
Ramachandran Outliers(%)	0
Average *B*-factor (Å^2)	38.58

†Highest resolution shell is shown in parenthesis.

Table S3. The sequences of primers for RT-qPCR, related to STAR Methods.

Primer	Sequence
**IFN-β1**	5′-AACTGCAACCTTTCGAAGCC-3′ (forward)<br>5′-TGTCGCCTACTACCTGTTGTGC-3′ (reverse)
**TNF-α**	5′-GAGGCCAAGCCCTGGTATG-3′ (forward)<br>5′-CGGGCCGATTGATCTCAGC-3′ (reverse)
**IL-6**	5′-CCAGGAGAAGATTCCAAAGATGTA-3′ (forward)<br>5′-CGTCGAGGATGTACCGAATTT-3′ (reverse)
**CCL5**	5′-TGCCCACATCAAGGAGTATTT-3′ (forward)<br>5′-GATGTACTCCCGAACCCATTT-3′ (reverse)
**CXCL2**	5′-GCTTGTCTCAACCCCGCATC-3′ (forward)<br>5′-TGGATTTGCCATTTTTCAGCATCTT-3′ (reverse)
**IFN-λ1**	5′-CCTCACGCGAGACCTCAAAT-3′ (forward)<br>5′-GAGTAGGGCTCAGCGCATAA-3′ (reverse)
**IFN-λ2**	5′-AATTGTGTTGCCAGTGGGGA-3′ (forward)<br>5′-GCGACTGGGTGGCAATAAAT-3′ (reverse)
**IFN-λ3**	5′-CCCAAAAAAGGAGTCCCCTG-3′ (forward)<br>5′-GGTTGCATGACTGGCGGA-3′ (reverse)
**GAPDH**	5′- GTCTCCTCTGACTTCAACAGCG-3′ (forward)<br>5′- ACCACCCTGTTGCTGTAGCCAA -3′ (reverse)
